# Bioinspired Nanomodification Strategies: Moving from Chemical-Based Agrosystems to Sustainable Agriculture

**DOI:** 10.1021/acsnano.1c03948

**Published:** 2021-08-04

**Authors:** Liang Xu, Zhiwei Zhu, Da-Wen Sun

**Affiliations:** † School of Food Science and Engineering, South China University of Technology, Guangzhou 510641, China; ‡ Academy of Contemporary Food Engineering, South China University of Technology, Guangzhou Higher Education Mega Center, Guangzhou 510006, China; § Engineering and Technological Research Centre of Guangdong Province on Intelligent Sensing and Process Control of Cold Chain Foods, & Guangdong Province Engineering Laboratory for Intelligent Cold Chain Logistics Equipment for Agricultural Products, Guangzhou Higher Education Mega Center, Guangzhou 510006, China; ∥ Food Refrigeration and Computerized Food Technology (FRCFT), Agriculture and Food Science Centre, University College Dublin, National University of Ireland, Belfield, Dublin 4, Ireland

**Keywords:** biosynthesis, nanoparticles, soil remediation, seed germination, heavy-metal stress, toxicity, antimicrobial activity, pest management

## Abstract

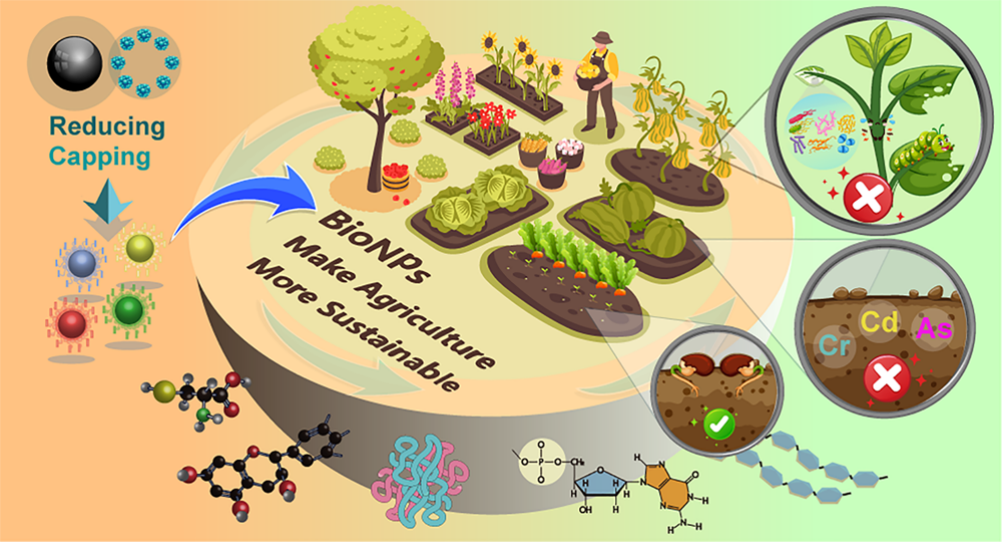

Agrochemicals have supported the development of the agricultural economy and national population over the past century. However, excessive applications of agrochemicals pose threats to the environment and human health. In the last decades, nanoparticles (NPs) have been a hot topic in many fields, especially in agriculture, because of their physicochemical properties. Nevertheless, the prevalent methods for fabricating NPs are uneconomical and involve toxic reagents, hindering their extensive applications in the agricultural sector. In contrast, inspired by biological exemplifications from microbes and plants, their extract and biomass can act as a reducing and capping agent to form NPs without any toxic reagents. NPs synthesized through these bioinspired routes are cost-effective, ecofriendly, and high performing. With the development of nanotechnology, biosynthetic NPs (bioNPs) have been proven to be a substitute strategy for agrochemicals and traditional NPs in heavy-metal remediation of soil, promotion of plant growth, and management of plant disease with less toxicity and higher performance. Therefore, bioinspired synthesis of NPs will be an inevitable trend for sustainable development in agricultural fields. This critical review will demonstrate the bioinspired synthesis of NPs and discuss the influence of bioNPs on agricultural soil, crop growth, and crop diseases compared to chemical NPs or agrochemicals.

Sustainable agriculture lays a solid foundation for a nation’s economic development, environmental protection, and food security.^1^ Crop diseases caused by plant pathogens and pests are the primary troubles for losses of crop yield and restriction of agricultural development.^2^ Agrochemical pesticides have controlled the pathogens and prevented agricultural yield losses successfully over the past century. However, excessive applications and misuses of pesticides bring about the emergence of drug resistance and make agrochemicals awkward.^3^ Also, agrochemicals cause environmental pollution and accumulate in nontarget living organisms, such as fish, beneficial microorganisms in the soil, honey bees, and earthworms. The pesticide accumulation poses a risk to biodiversity and threatens human health *via* the food chain eventually.^2^ Therefore, alternative methods to control the pathogens and pests in an ecofriendly manner are urgent for developing sustainable and intensified agriculture.

Nanoparticles (NPs) have been in the limelight of up-to-date nanotechnology owing to their special physical and chemical properties over the last decades.^4−9^ Some characteristics (Table 1), including the catalytic, superparamagnetic (iron oxide NPs), antimicrobial, and anticancer activity, render NPs versatile in many application fields like biomedical and pharmaceutical industries, wastewater treatment, remediation of environmental pollutants, and food storage.^10−15^ In particular, agriculture belongs to a significant area of the applications of NPs. Several scientists have reported different methods, *viz*., physical, chemical, and biological methods, to synthesize NPs.^12^ However, physical synthesis processes, including grinding, mechanical milling, laser ablation, and sputtering, are expensive and energy-consuming.^16^ Meanwhile, chemical methods include sol–gel, precipitation, thermal decomposition, microemulsion, hydrothermal, microwave irradiation, and colloidal thermal synthesis.^17^ These processes require expensive equipment and toxic reactants such as thiocarbamide, thiophenol, NH_2_OH, N_2_H_4_, and NaBH_4_.^18^ These techniques also generate several toxic byproducts and require nonbiodegradable capping agents for stabilization of the NPs. Considering the above limitations, more and more scientists pay attention to biological synthesis since it is the most ecofriendly, convenient, and economical approach to prepare NPs.

**Table 1:** Characteristics of Traditional NPs and BioNPs

characteristics of NPs	traditional NPs	bioinspired NPs
catalytic activity	NPs are efficient catalysts owing to their high surface-to-volume ratio, conductivity, and electrostatic attraction^177^	stronger catalytic activity^178^
superparamagnetic properties (iron oxide NPs)	contaminant removal in water and soil^179^	ecofriendly route for contaminant removal^82,180^
toxicity	adverse effect on environment and human health^72,181^	less toxicity to normal cells, including animal and plant cell^127,182,183^
antimicrobial activity	microbial death was induced by cell membrane destruction, ROS production, mitochondrial damage, protein dysfunction, and DNA damage^157,158^	stronger antimicrobial activity^19,184,185^
antioxidative activity	*–*	strong antioxidative activity^22,37,186^
anticancer activity	cancer cell death was induced by intracellular ROS production, mitochondrial dysfunction, and nuclear damage^187^	stronger anticancer activity^23^
stability	high surface energy of NPs results in aggregation and instability^188^	higher stability^21,189^

In the natural environment, microorganisms and plants can reduce metal ions to metal NPs by various bioactive substances to mitigate the toxicity of metal ions or utilize them as nutrients for growth.^15^ Natural active substances are widely accepted by researchers and the general public due to their practical, ecofriendly, and biocompatible attributes. Inspired by these biological exemplifications for the synthesis of NPs, increasing research focuses on the bioinspired synthesis of NPs rather than chemical synthesis. Bioinspired synthesis combines biological concepts, mechanisms, and functions for the design and development of bioderived (nano)materials with various applications.^15^ Furthermore, bioinspired synthesis possesses the following merits as compared with chemical synthesis: (a) Biosynthesis is facile and usually takes a one-pot reaction because bioactive substances can be employed as reducing and capping agents simultaneously; (b) biosynthesis is cost-effective and accessible to scale-up production due to cheap raw materials and simple processes; (c) biosynthesis can functionalize nanomaterials, boosting their stability and performance in various applications; and (d) biosynthesis without involving toxic and hazardous chemicals potentiates the biocompatibility of the resulting product with organic entities. In addition, bioinspired synthesis awards NPs more advantageous characteristics compared to traditional syntheses, such as stronger antimicrobial activity, less toxicity, higher stability, *etc.* This could be ascribed to the capping biomaterials from microbial or plant metabolites which possess effective antimicrobial activity and high biocompatibility.^19,20^ The higher stability could be afforded by electrostatic and steric interactions due to charged biomolecules adsorbed on the surface of bioNPs.^21^ Therefore, the nature of capping biomaterial plays an essential role in the property of bioNPs. For instance, the plant extract rich in polyphenol is utilized to synthesize NPs, enhancing the antioxidant activity of NPs.^22^ The cell-free filtrate from a microorganism, which can induce a pro-oxidant and cytotoxic effect on cancer cells, is attached to NPs surface, endowing NPs more effective anticancer activity.^23^ Over the past decade, bioNPs have exhibited extensive and positive chemical interactions to agricultural systems ranging from crop disease management, agricultural yield improvement to environmental safety. NPs such as silver (AgNPs), gold (AuNPs), copper (CuNPs), palladium (PdNPs), selenium (SeNPs), zinc oxide (ZnONPs), magnesium oxide (MgONPs), titanium dioxide (TiO_2_NPs), and iron oxide NPs, *etc.* have proven to protect the plant from infection by bacteria, fungi as well as pests. Apart from being a crop disease treatment, the bioNPs can become promoters for seed germination and plant growth, improving the crop yield. Also, the bioNPs can remediate the contaminated soil with less ecotoxic compared with commercial NPs. Therefore, bioinspired synthesis of NPs is an inevitable trend in ecofriendly nanotechnology, driving nanotechnology to enhance the sustainability of agricultural production.

In recent years, many reviews have summarized the fundamental applications of bioNPs in environmental remediation,^11,13,16^ biomedicine,^14,24^ and food storage fields.^10^ However, few comprehensive reviews discuss the up-to-date roles of bioNPs in sustainable agriculture. Recent reviews have touched upon this topic.^25,26^ One of them comprehensively reviewed the antimicrobial activity of bioNPs against plant pathogens and clarification the phytotoxicity of bioNPs.^25^ Another systematically summarized the microbial synthesis of NPs and their beneficial agricultural applications as biosensors, pesticides, and fertilizers.^26^ Different from the previous reviews, this contribution intends to comprehensively overview the advances in applications and mechanisms of bioNPs in the agricultural system, ranging from heavy-metal remediation in soil, seed germination, crop growth, resistance to heavy-metal stress, and phytotoxicity to crop diseases and pest attacks management, over the past 10 years and elaborate the future trends. This review consists of five parts (Figure 1), in which (a) bioinspired synthesis of NPs, (b) bioNPs for agricultural soil, (c) bioNPs for crop growth, and (d) bioNPs for crop disease management are illustrated, and (e) further prospects, challenges, and current-stage conclusions are discussed. Despite some infancy-stage applications, the bioNPs have been demonstrated to be a beneficial and leading-edge solution to sustainable agriculture.

**Figure 1:**
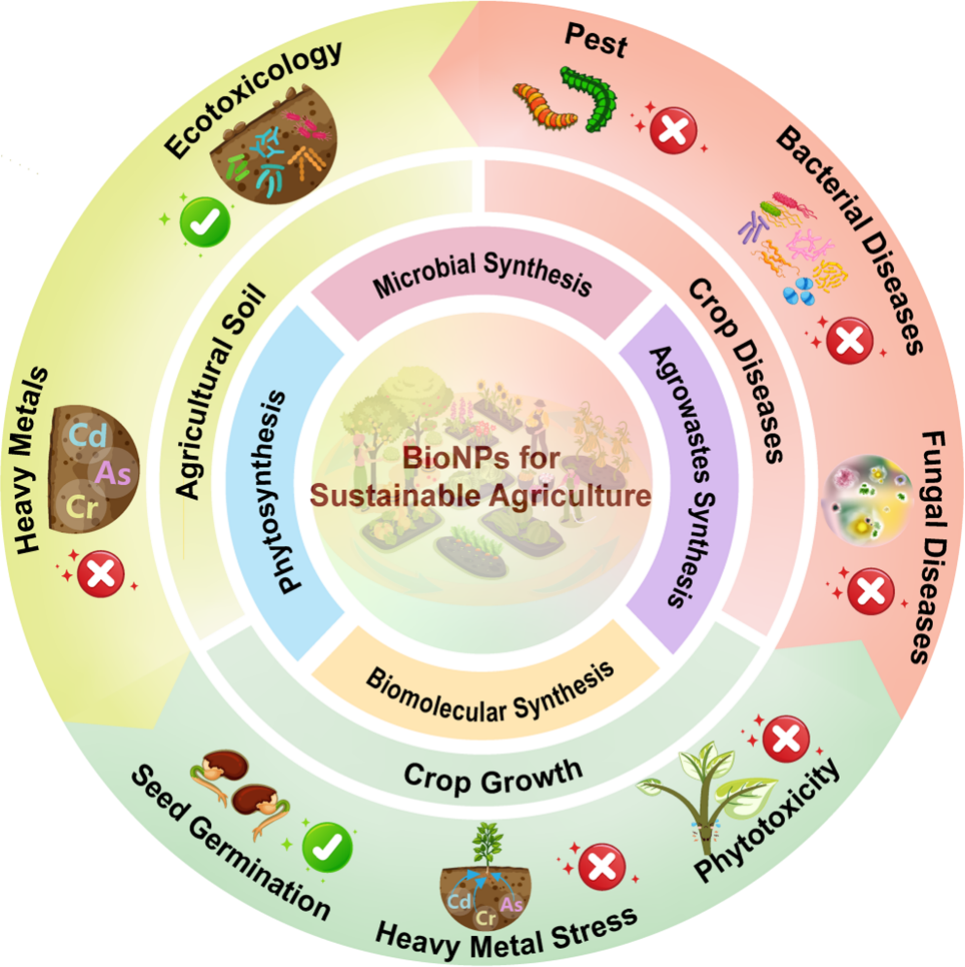
Schematic representation of bioNPs synthesis and applications of bioNPs in agricultural soil, crop growth, and crop diseases.

## Bioinspired Synthesis of BioNPs

Bioinspired synthesis of NPs can be economical and environmentally friendly compared with chemical synthesis, attracting much attention from researchers in recent years. Microorganisms, plants, algae, and biomolecules are excellent candidates for producing bioNPs. The bioinspired synthesis of various NPs is summarized in Table 2. In general, the range of synthetic temperature is between room temperature and 40 °C and pH adjustment is unnecessary. These mild synthetic conditions are based on the optimal cultural conditions of microorganisms or extraction conditions of biomolecules. It is believed that the microbial physiological function and biomolecular activity, including metal ions bonding and reductive activity, play a crucial role in biosynthetic efficiency. In addition, the reductive agents applied for synthesis are derived from biological materials, providing an ecofriendly avenue of advanced nanotechnology to overcome synthetic challenges. This section aims to discuss the advances in the bioinspired synthesis of NPs.

**Table 2:** Bioinspired Synthesis of Various NPs

s. no.	species	types of NPs	size (nm) and shape	incubation temperature and time	reducing agents	synthetic temperature, pH, time	ref
Bacteria							
1	*Bacillus amyloliquefaciens*	Ag	20–40, spherical	37 °C, 24 h	cell-free supernatant	*T*_room_, –a, 48 h	(190)
2	*Bacillus brevis* (NCIM 2533)	Ag	41–68, spherical	37 °C, 24 h	cell-free supernatant	*T*_room_, –a, overnight	(191)
3	*Bacillus flexus*	Ag	12–61, spherical and triangular	37 °C, 24 h	cell-free supernatant	*T*_room_, –a, 8 h	(192)
4	*Bacillus licheniformis* Dahb1	Ag	19–63, spherical	37 °C, 24 h	cell-free supernatant	*T*_room_, –a, 24 h	(193)
5	*Bacillus marisflavi* TEZ7	Ag	11–39, spherical	28 °C, 24 h	cell-free supernatant	28 °C, –a, 24 h	(194)
6	*Bacillus safensis* TEN12	Ag	23–46, spherical	28 °C, 24 h	culture medium	28 °C, –a, 24 h	(182)
7	*Bacillus subtillis*	Ag	∼59, spherical	33 °C, 48 h	cell-free supernatant	33 °C, –a, 48 h	(195)
8	*Bacillus thuringiensis*	Ag	10–30, spherical	37 °C, 24 h	culture medium	37 °C, –a, 24 h	(196)
9	*Pseudomonas fluorescens* PMMD3	Ag	1–10, spherical	30 °C, 24 h	cell-free supernatant	30 °C, -a, 6 h	(197)
10	*Pseudomonas aeruginosa*	Ag	∼80, spherical	37 °C, 24 h	cell-free supernatant	37 °C, –a, 72 h	(198)
11	*Paracoccus haeundaensis* BC74171^T^	Au	∼21, spherical	25 °C, 48 h	cell-free supernatant	70 °C, –a, 15 min	(186)
12	*Vibrio alginolyticus*	Au	50–100, irregular	40 °C, 24 h	cell-free supernatant	40 °C, –a, 24 h	(199)
13	*Bacillus marisflavi* YCIS MN 5	Au	∼14, spherical	*T*_room_, 24 h	cell-free supernatant	*T*_room_, –a, 96 h	(200)
14	*Shigella flexneri* SNT22	Cu	17–38, spherical	*T*_room_, overnight	culture medium	30 °C, –a, 24 h	(109)
15	*Escherichia sp.* SINT7	Cu	∼29, spherical	28 °C, 24 h	culture medium	28 °C, –a, 24 h	(201)
16	*Klebsiella pneumoniae*	Cu	19–47, spherical	30 °C, 24 h	culture medium	30 °C, –a, 24 h	(108)
17	*Morganella morganii*	Cu	15–20, quasi-spherical	37 °C, 24 h	culture medium	37 °C, –a, 20 h	(30)
18	*Morganella psychrotolerans*	Cu	4–60, irregular	20 °C, 24 h	washed cells	20 °C, –a, 24 h	(185)
19	*Shewanella loihica* PV-4	Cu	10–16, spherical	30 °C, 24 h	washed cells	30 °C, –a, 120 h	(202)
20	*Burkholderia rinojensis*	MgO	∼27, spherical	37 °C, 24 h	washed cells	*T*_room_, –a, 10 h	(136)
21	*Acinetobacter johnsonii* RTN1	MgO	18–45, spherical	28 °C, overnight	cell-free supernatant	28 °C, –a, 24 h	(150)
22	*Bacillus* sp. RNT3	MgO	22–52, spherical	28 °C, 24 h	cell-free supernatant	28 °C, –a, 24 h	(149)
23	*Shewanella loihica* PV-4	Pd	1–20, spherical	30 °C, 24 h	washed cells	30 °C, –a, 72 h	(79)
24	*Geobacter sulfurreducens*	Pd	∼14, spherical	30 °C	washed cells	30 °C, –a, 24 h	(203)
25	*Stenotrophomonas maltophilia* SeITE02	Se	160–250, spherical	27 °C, 48 h	culture medium	27 °C, –a, 48 h	(204)
26	*Bacillus amyloliquefaciens*	TiO_2_	22–97, spherical	37 °C, 96 h	culture medium	37 °C, –a, 24 h	(205)
27	*Bacillus thuringiensis*	ZnO	15–25, hexagonal	37 °C, 48 h	culture medium	37 °C, –a, 12 h	(172)
Fungi							
1	*Macrophomina phaseolina*	Ag	5–40, spherical	28 °C, 8 d	cell-free filtrate	28 °C, –a, 72 h	(206)
2	*Setosphaeria rostrata*	Ag	2–20, spherical	28 °C, 7 d	cell-free filtrate	*T*_room_, –a, 24 h	(207)
3	*Trichoderma harzianum*	Ag	∼51, spherical	27 °C, 5 d	cell-free filtrate	40 °C, –a, 5 h	(208)
4	*Trichoderma viride* (MTCC 5661)	Ag	10–20, spherical	28 °C, 7 d	cell-free filtrate	28 °C, –a, 16 h	(19)
5	*Penicillium janthinellum* DJP06	Ag	1–30, quasi-spherical	28 °C, 6 d	cell-free filtrate	28 °C, –a, 72 h	(209)
6	*Fusarium oxysporum*	Ag	∼21, spherical	25 °C, 4 d	cell-free filtrate	25 °C, –a, 48 h	(210)
7	*Penicillium cyclopium*	Ag	12–25, irregular	*T*_room_, 4 d	washed mycelium	25 °C, –a, 24 h	(34)
8	*Rhizopus oryzae*	Ag	∼7, spherical	30 °C, 3 d	cell-free filtrate	30 °C, –a, 24 h	(211)
9	*Aspergillus niger*	Ag	20–60, spherical	28 °C, 5 d	cell-free filtrate	28 °C, –a, 24 h	(184)
10	*Cladosporium oxysporum* AJP03	Au	∼72, quasi-spherical	28 °C, 6 d	cell-free filtrate	28 °C, –a, 120 h	(178)
11	*Trichoderma harzianum*	Au	26–34, spherical	28 °C, 5 d	washed mycelium	28 °C, –a, 72 h	(32)
12	*Trichoderma harzianum*	Au	∼30, spherical	30 °C, 3 d	washed mycelium	30 °C, –a, 10 h	(212)
13	*Penicillium expansum*	γ-Fe_2_O_3_	15–66, spherical	30 °C, 7 d	cell-free filtrate	*T*_room_, –a, overnight	(213)
14	*Aspergillus niger* BSC-1	Fe_3_O_4_	20–40, flake like	28 °C, 15 d	cell-free filtrate	28 °C, –a, 3 h	(82)
15	*Fusarium oxysporum*	ZnO	18–25, irregular	27 °C, 3 d	cell-free filtrate	80 °C, –a, 3 h	(214)
16	*Aspergillus sp.* NJP02	ZnO	80–120, quasi-spherical	28 °C, 6 d	cell-free filtrate	28 °C, –a, 72 h	(215)
17	*Aspergillus fumigatus* TFR-8	ZnO	∼22, quasi-spherical	28 °C, 5 d	cell-free filtrate	28 °C, –a, 72 h	(101)
18	*Cochliobolus geniculatus*	ZnO	2–6, quasi-spherical	28 °C, 6 d	cell-free filtrate	28 °C, –a, 72 h	(33)
19	*Trichoderma* sp. WL-Go	Se	20–220, quasi-spherical	28 °C, 2 d	culture medium	30 °C, –a, 48 h	(216)
Microalgae							
1	*Chlorella pyrenoidosa*	Ag	5–20, irregular	24 °C, 22 d	algal cell extract	28 °C, –a, 24 h	(217)
2	*Trichodesmium erythraeum*	Ag	27, cubical	–	algal cell extract	*T*_room_, –a, 24 h	(218)
3	*Nostoc muscorum* NCCU-442	Ag	6–45, spherical	30 °C	algal cell extract	30 °C, –a, 24 h	(219)
4	*Spirulina platensis*	Ag	∼29, spherical	37 °C, 24 d	methanolic extract	*T*_room_, –a, 20 min	(220)
5	*Acutodesmus dimorphus*	Ag	2–20, spherical	35 °C, 8 dpart	algal cell extract	*T*_room_, –a, 24 h	(221)

s. no.	species	types of NPs	size (nm) and shape	part	reducing agents	synthetic temperature, pH, time	ref
Plants							
1	*Diplazium esculentum (retz.)* sw.	Ag	10–45, spherical, oval and triangular	leaf	aqueous extract	*T*_room_, –a, 12 h	(222)
2	*Convolvulus arvensis*	Ag	10–30, spherical	leaf	aqueous extract	*T*_room_, 9, 2 h	(223)
3	*Alpinia nigra*	Ag	∼6, spherical	fruit	aqueous extract	*T*_room_, –a, 1 h	(224)
4	Longan	Ag	4–10, spherical	fruit	aqueous extract	*T*_room_, –a, 80 min	(225)
5	*Acacia nilotica*	Ag	20–30, spherical	pod	aqueous extract	*T*_room_, 9, 24 h	(226)
6	*Jasmine*	Ag	∼40, fiber shaped	flower	aqueous extract	*T*_room_, –a, 30 min	(227)
7	*Moringa oleifera*	Ag	∼8, spherical	flower	aqueous extract	*T*_room_, –a, 30 min	(228)
8	*Salacia chinensis*	Ag	40–80, spherical	bark	aqueous extract	*T*_room_, –a, 4 h	(229)
9	*Ocimum basilicum*	Ag	∼14, crystalline	seed	aqueous extract	*T*_room_, –a, 6 h	(230)
10	*Zinnia elegans*	Au	∼25, spherical	leaf	ethanolic extract	*T*_room_, –a, 1.25 h	(231)
11	*Glycyrrhzia glabra* L.	Au	3–16, circular	root	ethanolic extract	25 °C, 5, 2.5 h	(232)
12	*Taraxacum laevigatum*	Pt	2–7, spherical	leaf	aqueous extract	90 °C, –a, 10 min	(233)
13	*Prosopis juliflora*	ZnO	∼65, spherical	leaf	aqueous extract	170 °C, –a, 5 h	(234)
14	*Eriobutria japonica*	ZnO	∼50, hexagonal	seed	aqueous extract	60 °C, 12, 2 h	(235)
15	Quince	ZnO	∼25, crystalline	seed	mucilage extract	80 °C, –a, 2 h	(236)
16	*Jatropha curcas* L.	TiO_2_	10–20, spherical	leaf	aqueous extract	*T*_room_, –a, 20 min	(237)
17	*Parthenocissus quinquefolia*	Fe	50–80, rounded	leaf	aqueous extract	*T*_room_, –a, 24 h	(238)
18	Cinnamomum Verum	Fe	20–50, circular and spherical	bark	aqueous extract	*T*_room_, –a, 24 h	(239)
19	*Rumex acetosa*	Fe_*x*_O_*y*_	10–40, spherical	leaf	aqueous extract	*T*_room_, 4, 30 min	(189)
20	*Excoecaria cochinchinensis*	Fe_3_O_4_	20–30, spherical	leaf	aqueous extract	70 °C, –a, 2 h	(180)
21	*Euphorbia cochinchinensis*	Fe_3_O_4_	10–30, spherical	leaf	aqueous extract	70 °C, –a, 2 h	(240)
22	*Commelina nudiflora*	Cu	45–100, spherical	whole	aqueous extract	45 °C, 9, 6 h	(241)
23	*Ziziphus spina-christi* L. Willd.	Cu	5–20, spherical	fruit	aqueous extract	80 °C, –a, –a	(242)
24	*Rosa canina*	CuO	15–25, spherical	fruit	aqueous extract	100 °C, –a, 1 h	(243)
25	*Fortunella japonica*	CuO	5–10, spherical	fruit	aqueous extract	90 °C, 10, 1 h	(130)
26	Jujube	SnO_2_	∼18, crystalline	fruit	aqueous extract	*T*_room_, –a, 30 min	(244)
27	*Aegle marmelos*	NiO	8–10, spherical	leaf	aqueous extract	250 °C, –a, 15 min	(245)
28	*Solanum trilobatum*	NiO	∼23, cylindrical and rod-like	leaf	aqueous extract	250 °C, –a, 15 min	(246)
29	Moso bamboo	MnO_*x*_	30–80, cuboid	whole	ethanolic extract	*T*_room_, –a, 24 h	(247)
30	*Vernonia amygdalina*	MnO_2_	20–22, flower like	leaf	aqueous extract	*T*_room_, 6, 105 min	(248)
31	*Amaranthus tricolor*, *Andrographis paniculata*, or *Amaranthus blitum*	MgO	18–80, spherical	leaf	aqueous extract	60 °C, –a, 10 min	(249)
32	*Costus pictus* D. Don	MgO	∼50, hexagonal	Leaf	Aqueous extract	80 °C, –a, 4 h	(250)
Seaweed							
1	*Padina gymnospora*	Ag	2–20, spherical	whole	methanolic extract	100 °C, –a, 2 h	(251)
2	*Gracilaria birdiae*	Ag	20–95, spherical	whole	ethanolic extract	90 °C, 10–11, 30 min	(252)
3	*Gracilaria verrucosa*	Au	20–80, spherical and triangular	whole	aqueous extract	60 °C, 7, 30 min	(253)
4	*Padina gymnospora*	Pt	∼25, truncated octahedral	whole	aqueous extract	*T*_room_, –a, 10 min	(254)
5	*Ulva lactuca*	ZnO	10–50, triangle, hexagonal, rectangle, and rod like	whole	aqueous extract	70 °C, –a, 4 h	(255)
6	*Padina tetrastromatica*	ZnO	28, hexagonal	whole	aqueous extract	80 °C, –a, 2 h	(256)
7	*Sargassum myriocystum*	ZnO	96–110, rectangle, spherical, triangle, and radial	whole	aqueous extract	80 °C, 8, 10 min	(257)
Biomolecules							
1	cysteine	Ag	8–18, spherical	amino acid	aqueous extract	24 °C, –a, 2 d	(52)
2	cysteine	Ag	∼13, spherical	amino acid	aqueous extract	60 °C, 9, 5 h	(53)
3	tyrosine	Ag	13–33, irregular	amino acid	aqueous extract	90 °C, 10–12, 30 min	(258)
4	keratinase	Ag	3–15, spherical	protein	aqueous extract	37 °C, –a, 48 h	(47)
5	bovine serum albumin	Au	∼4, spherical	protein	aqueous extract	37 °C, –a, 12 h	(48)
6	laccase	Au	71–266, spherical	protein	aqueous extract	70 °C, –a, 20 min	(45)
7	lignin peroxidase	Au	∼10, spherical	protein	aqueous extract	37 °C, –a, 10 h	(46)
8	α-amylase enzyme	ZnO	∼11, spherical	protein	aqueous extract	25 °C, –a, 2 h	(94)
9	quercetin	Pd	100–300, spherical	phenolic	aqueous extract	*T*_room_, –a, 5 h	(259)
10	*Lactobacillus brevis*	Ag	30–100, spherical	polysaccharide	aqueous extract	*T*_room_, –a, 60 min	(260)
11	xanthan gum	Ag	8–40, spherical	polysaccharide	aqueous extract	80 °C, –a, 2 h	(261)
12	pectin	Ag	8–28, spherical	polysaccharide	aqueous extract	*T*_room_, –a, 24 h	(262)
13	*Chlorella vulgaris*	Ag	4–9, spherical	polysaccharide	aqueous extract	85 °C, 10, 20 min	(263)
14	arabinoxylan	Ag	∼25, spherical	polysaccharide	aqueous extract	75 °C, –a, 90 min	(264)
15	*Aegle marmelos gum*	Au	∼92, triangular	polysaccharide	aqueous extract	70 °C, –a, 2 h	(265)
16	levan	Au	10–12, spherical	polysaccharide	aqueous extract	100 °C, –a, 30 min	(266)
17	lignin	Cu	50–150, needle like	polysaccharide	aqueous extract	*T*_room_, –a, 2 h	(267)
18	β-d-glucans	Au	∼30, quasi-spherical	saccharide	aqueous extract	90 °C, 7, 3 h	(268)
Agrowastes							
1	red onion	Ag	∼12.5, spherical	peel	aqueous extract	90 °C, –a, 30 min	(269)
2	mango	Ag	7–27, crystalline	peel	aqueous extract	80 °C, 11, 15 min	(270)
3	grape	Ag	3–14, crystalline and spherical	pomace	aqueous extract	90 °C, –a, 20 min	(271)
4	*Nypa fruticans*	Au	15–20, spherical	husk	aqueous extract	25 °C, –a, 20 min	(272)
5	*Zea maysL.*	CuO	36–73, quasi-spherical and conical	husk	aqueous extract	70–80 °C, 4, 2 h	(273)
6	papaya	Pd	1–5, spherical	peel	aqueous extract	*T*_room_, –a, 2 d	(274)
7	banana	ZnO	20–40, flower-like and cubic	peel	aqueous extract	*T*_room_, 12, 2 h	(275)
8	*Citrus sinensis*	ZnO	∼33, hexagonal	peel	aqueous extract	*T*_room_, –a, 10 min	(276)
9	*Phoenix dactylifera*	ZnO	∼30, spherical	date pulp waste	aqueous extract	*T*_room_, –a, 30 min	(277)

a Not mentioned in the corresponding reference.

### Microbial Synthesis of BioNPs

Microorganisms, including bacteria and fungi, can synthesize NPs extracellularly or intracellularly through cultivation in a medium during an incubation time. These creatures mitigate the toxicity of noble metal ions or utilize some metal ions as their nutrition for growing through reducing the metal ions to metal NPs by various metal ion reductases. These reductases or other relevant proteins become a layer (corona) to cover the surface of NPs, awarding bioNPs more substantial functionality and stability.

**Figure 2:**
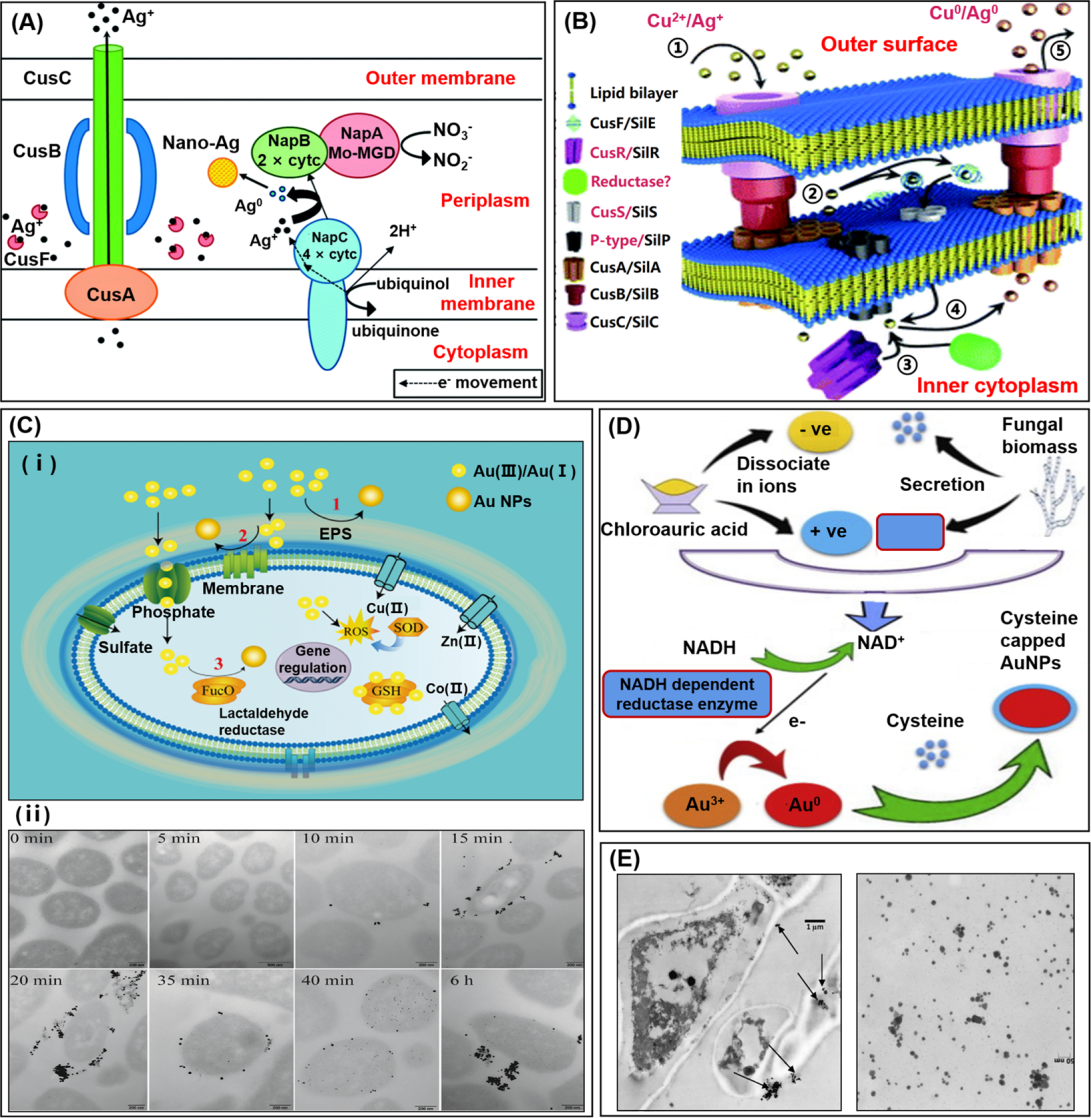
Schematic illustration of microbial synthetic mechanisms of bioNPs. (A) Schematic representation of AgNPs biosynthesis by periplasmic c-type cytochrome NapC in the silver-resistant *E. coli.* The Ag^+^ are excluded by the CusCBA from the cytoplasm and concentrated in the periplasmic space. Reproduced with permission from ref (27). Copyright 2014 Royal Society of Chemistry. (B) A mode of the similarities between the copper and silver resistance systems. The process involves the cellular uptake of Cu^2+^ (step 1) followed by the silver resistance machinery (step 2). Metal ion reductases bind to Cu^2+^ (step 3), reducing Cu^2+^ to CuNPs (step 4). These NPs are released from the cell using a cellular efflux system (step 5). Reproduced with permission from ref (30). Copyright 2013 Royal Society of Chemistry. (C) (i) Au(III) reduction outside the cell (part 1), Au(III) reduction on the cell wall (part 2), and enzymatic reduction in the cytoplasm (part 3). (ii) TEM images of thin sections of cells incubated in Au(III) for different times: The first appearance of NPs was detected in the culture medium at 10 min. Afterward, the NPs were monitored on the cell wall at 15 min and observed in the cytoplasm at 35 min. Reproduced with permission from ref (31). Copyright 2018 Royal Society of Chemistry. (D) Mechanism for AuNPs biosynthesis by *T. harzianum*. Reproduced with permission from ref (32). Copyright 2014 Elsevier. (E) TEM images of AgNPs (arrows) synthesized by the mycelium (left) and the cell-free extract (right) of *P. cyclopium.* Reproduced with permission from ref (34). Copyright 2019 Elsevier.

Lin *et al*. used a silver-resistant 116AR *Escherichia coli* strain to produce AgNPs successfully. However, a silver-sensitive 116S *E. coli* strain was rapidly inactivated after exposure to AgNO_3_, and no AgNPs were obtained, implying that the biosynthesis of AgNPs demands bacteria with a silver-resistant attribute.^27^ The silver-resistant bacteria bear glutathione or cysteine-containing polypeptides/proteins that can strongly interact with and neutralize Ag^+^ ion to mitigate the toxicity of Ag^+^ ion. As shown in Figure 2A, *E. coli* possess various *c*-type cytochromes in the inner membrane like NapC and the periplasm like NapA and NapB.^27^ The NapC is responsible for transferring the electron from the membrane-bound menaquinol to periplasmic NapA and NapB. These *c*-type cytochromes mediate electron transfer in the reduction of nitrate or nitrite under anaerobic conditions.^28^ In addition, Ramanathan *et al*. executed linear sweep voltammetry (LSV) experiments on silver-resistant bacteria, *Morganella psychrotolerans*, with Ag^+^ ions solution to study the mechanism of Ag^+^ ion reduction within the bacteria.^29^ They proposed the mechanism that the bacterial cells first took up the Ag^+^ ions after interacting with proteins within the cells, wherein they experienced an intracellular reduction to form AgNPs, and eventually pumped the AgNPs out of the cells. The extracellular proteins from *M. psychrotolerans* were extracted to reduce Ag^+^ ions, and a noticeably lower synthesized rate was obtained, proving that bacterial physiological function plays a crucial role in synthesizing AgNPs rather than extracellular proteins only. It could be easily deduced that a deviation of optimum culture conditions would negatively impact the bacterial physiological function, including metal ions adsorption and reductive abilities, and biosynthetic efficiency of NPs. Two years later, Ramanathan *et al*. published another relevant research regarding the biosynthesis of CuNPs using *Morganella morganii* RP42.^30^ The periplasmic silver binding proteins (SilE) showed 47% homology in the protein sequences with Cu^2+^ binding proteins (CusF), which has been well studied in copper-resistant systems from *E. coli* previously. This finding implied that SilE might have a similar ability to take up Cu^2+^ ions with CusF. It is worth noting that proteins/peptides containing a large number of histidine residues could strongly interact with Cu^2+^ ions and the SilE is rich in histidine, empowering *Morganella* sp. the abilities to take up Ag^+^ ions and Cu^2+^ ions. Besides, SilA, SilB, and SilC also play primary roles in taking up Cu^2+^ ions from solutions into bacterial cells, and SilS are responsible for sensing the Cu^2+^ ions (Figure 2B).

More importantly, Liu *et al*. provided insights into the dynamic process of AuNPs biosynthesis, and mechanisms for AuNP formation in *Pantoea* sp. IMH were proposed (Figure 2C(i)).^31^ They prepared AuNPs by culturing *Pantoea* sp. IMH with HAuCl_4_ for 12 h and monitored the course of NPs formation by TEM (Figure 2C(ii)). Their results showed that the reduction of NPs took place in the medium initially and in the cytoplasm finally. In extracellular synthesis, the acetal and hemiacetal groups in extracellular polymeric substances (EPS) perform as the reducing agent for Au(III) reduction. The EPS adhering to the cell surface reduce Au(III) to AuNPs, causing the distribution of AuNPs on the cell surface. In intracellular synthesis, the fucO protein can catalyze l-lactaldehyde into l-1,2-propanediol, which are the vital factors in reducing metal ions. After 12 h culture, the decreasing sizes of NPs from medium to cytoplasm were observed.^31^ The ∼50 nm NPs were dispersed in the medium, the ∼20 nm NPs were attached to the cell walls, and the ∼10 nm NPs were distributed in the cytoplasm. The NPs with protein fractions in the extracellular solution were aggregated, leading to the growth of NPs and a larger size. In contrast, the limited mobility inhibited NPs growth due to the high viscosity of the fluid in the cytoplasm.

Along with bacteria, fungi also possess metal-ion resistance mechanisms through secreting sulfur-containing proteins to mitigate the toxicity of metal ions. Furthermore, the reductase enzyme or other metabolites produced by fungi plays essential roles in reducing metal ions into metal NPs. Meanwhile, the as-synthesized bioNPs are encapsulated by various biomolecules such as proteins, organic acids, and polysaccharides, enhancing the performance and stability of NPs. In this regard, the properties of microbial-derived NPs are primarily dependent on the type of microorganism, and the identification of reducing and capping agent from fungi is thus vital. Tripathi *et al*. proposed a potential mechanism of AuNPs synthesis by fungal biomass (*Trichoderma harzianum*) (Figure 2D), showing that the NADH-dependent reductase enzymes from *T. harzianum* were responsible for reducing Au^+^ into Au^0^ and the cysteine performed as a capping agent, rendering AuNPs stable.^32^ Apart from mycelium, cell-free filtrate from fungal culture rich in active metabolites can effectively synthesize bioNPs. Kumari *et al*. prepared AgNPs utilizing cell-free filtrate of *Trichoderma viride* and the AgNPs was characterized by gas chromatography–mass spectroscopy (GC-MS).^19^ They found that 16 metabolites, such as amino acids, sugars, organic acids, *etc*., contributed to the capping of AgNPs and some of them were potent antimicrobial agents, potentiating the antimicrobial activity of AgNPs. Similarly, the cell-free filtrate from *Cochliobolus geniculatus* was purified for the SDS-PAGE analysis to identify the critical protein in ZnONPs biosynthesis.^33^ The protein fractions of 97–36 kDa were attributed to reducing zinc acetate into ZnONPs, and protein fractions of 58 and 52 kDa were involved in capping ZnONPs. Another impressive work has been done by Wanarska *et al*., who investigated the AgNPs synthesis by both the mycelium and cell-free filtrate from *Penicillium cyclopium*.^34^ The left TEM images showed that the synthesized AgNPs were localized on the cell wall, indicating that the Ag^+^ reduction and AgNPs formation took place on the mycelium surface (Figure 2E). These results could be ascribed to the negatively charged surface of fungi due to the anionic structure tending to bind metal cations. The right TEM images in Figure 2E recorded that the AgNPs were irregular in shape and aggregated into larger particles. The saccharides and proteins in the cell wall were responsible for the synthesis by mycelium, while a protein with a molecular weight of 5 kDa involved the synthesis by cell-free filtrate, suggesting that the critical molecules for NPs synthesis were different by different approaches. In general, NPs can be biosynthesized by living or dead fungal cells, and microorganisms are an excellent factory to produce bioNPs for various applications. Nevertheless, the relatively long incubation period of fungal biomass prior to synthesis might restrict the application of fungal-developed NPs compared with bacterial synthesis.

### Phytosynthesis of BioNPs

Plant-mediated synthesis of NPs is widely investigated compared with microbial synthesis because the synthesis can be performed quickly and without cell incubation. Plant root, leaf, flower, and fruit extract, containing polyphenols, flavonoids, phenolic acids, vitamins, terpenoids, and alkaloids, are another practical resource for preparing various NPs. These compounds serve as a reducing and capping agent, directing the crystal growth and stabilizing the particle with a specific size by balancing the electrostatic force. It is worth noting that several synthetic parameters, such as the type of extraction solvent, reaction time, pH, temperature, and the concentration ratio of the plant extract and precursor metal ion, play significant roles in regulating the size and shape of NPs. The size, shape, and functional groups covered on the surface substantially affect the performance of bioNPs. Therefore, investigations for the optimization and mechanism of NPs phytosynthesis are necessary. Because the component of plant extracts is complicated, the precise mechanism of the NPs phytosynthesis is still not well understood. Zhang *et al*. synthesized AgNPs using cucumber leaf extract and identified 245 metabolites in the extract by GC-MS, screening out the key components responsible for AgNPs phytosynthesis.^35^ They found that some organic acids and specific reducing sugars, such as cellobiose, fructose, ribose, *etc*., noticeably were consumed during synthesis, suggesting that these metabolites were responsible for AgNPs formation. The organic acids containing −OH and −COOH groups were involved in reducing and stabilizing AgNPs.^36^ In respect to synthetic condition, Yousaf *et al*. utilized aqueous, ethanol, or methanol extracts of *A. millefolium* to obtain different sizes and shapes of AgNPs and found that the methanol extract possessed the highest antioxidant activity and the smallest AgNPs were synthesized compared with ethanol or aqueous extracts.^37^ This finding could be due to the different types of extraction solvent resulting in different components and quantity of extract. Rufus *et al*. investigated the effect of plant extract concentration on the size of Fe_2_O_3_NPs and their results exhibited that a higher concentration of plant extract led to smaller sizes of NPs.^38^ Yang *et al*. applied four different fruit juices to synthesize highly stable and ultrasmall AuNPs by regulating the solution pH and found that the high pH could decrease the size of AuNPs and prevent their aggregation, and these fruit-developed AuNPs were stable at room temperature for four months.^39^ The phytosynthesis of bioNPs provides a more facile and efficient protocol for the size and shape control compared with microbial synthesis. However, the extraction and purification of plant biomass can be a requisite challenge for the phytosynthesis of bioNPs.

### Biomolecular Synthesis of bioNPs

Biomolecules including phenolic compounds, polysaccharides, proteins, amino acids, and nucleic acid can serve as reducing and capping agents to prepare NPs. The phenolic compounds are usually derived from plants. The chemical structure of at least one hydroxyl group in a benzene ring is identified as phenolic compounds, which are divided into flavonoids and nonflavonoids. Flavonoids consist of two aromatic groups (A- and B-rings) linked by an oxygenated heterocyclic group (C-ring) to form the typical C_6_–C_3_–C_6_ skeleton (15 carbons in total number). These phenolic compounds are an ideal biomaterial for NPs synthesis due to their robust reductive and antioxidant activities. Myricetin, as a natural dietary flavonoid, was applied to synthesize AgNPs with sizes ranging from 20 to 50 nm.^40^ Podstawczyk *et al*. proposed an approach for the size-controllable synthesis of CuNPs using catechin and showed that the diameter of CuNPs increased with the pH value decreased.^41^ In an acid environment, the protonation of hydroxyl groups constrained the interaction between catechin and CuNPs, resulting in an enhanced agglomeration and larger diameters of CuNPs, whereas the deprotonation promoted the interaction with CuNPs surface in the alkaline environment, preventing CuNPs from agglomeration.

Apart from phenolic compounds, polysaccharides are an essential element for NPs fabrication, and polysaccharides can be procured by purifying from microorganisms and plants sources. There are many polysaccharides, including exopolysaccharides, starch, lignin, chitosan, cellulose, gum, *etc*. These compounds consist of hydroxyl groups, a hemiacetal reducing end, and other functional groups responsible for the reduction and stabilization of NPs. Exopolysaccharides (EPSs) are secreted by microorganisms and can be extracted from the cultural supernatant. Sathiyanarayanan *et al*. applied EPSs from *Bacillus subtilis* to synthesize spherical-shaped AgNPs in diameter of 60 nm, and the AgNPs were stable for five months.^42^ Interestingly, Li *et al*. found that the EPSs produced by *Lactobacillus plantarum* could self-assemble to become nanosize particles with a highly negative charge, possessing a strong adsorption capacity of heavy-metal ions.^43^ These EPSs were able to reduce HAuCl_4_ and AgNO_3_ to AuNPs and AgNPs, respectively, due to the hydroxyl groups and the electrostatic interactions of the amino group in EPS with AuCl_4_^–^ and sulfonic group with Ag^+^. In addition, three types of starch, including corn, cassava, and sago starch, were employed to synthesize AgNPs.^44^ The starch was mainly composed of amylose and amylopectin that could be hydrolyzed into glucose in hot water (40 °C), providing abundant hydroxyl groups to reduce Ag ions. Meanwhile, the carboxyl and hydroxyl groups of glucose and hydrolyzed starch could stabilize AgNPs to prevent aggregation.

Some enzymes, such as laccase, keratinase, peroxidases, fibrinolytic enzyme proteases, and reductases, can be applied in NPs synthesis, which are available by chromatographic purification of microbial cultural solution. For instance, the biosynthesis of AuNPs was supported by the purified laccase from *Paraconiothyrium variabile*.^45^ The lignin peroxidase from *Acinetobacter* sp. SW30 was applied for AuNPs synthesis.^46^ Keratinase from genetically modified bacteria *Bacillus subtilis* presented a relatively robust reducing capability, converting Ag^+^ into AgNPs.^47^ Besides, proteins, polypeptides, and amino acids possess strong interaction with metal ions and can be utilized in NPs synthesis. Bovine serum albumin was employed in the biosynthesis of NPs, such as AuNPs^48^ and RuO_2_NPs.^49^ When dissolving in a surfactant solution, the bovine serum albumin and zein protein were unfolded, exposing the reducing amino acids like cysteine, which could act as a reducing agent to reduce Au(III) to AuNPs.^50,51^ Cysteine in the bacterial cell can interact with Ag ions and mitigate the toxicity of Ag ions. Several researches revealed that cysteine could perform as a reducing and capping agent to synthesize AgNPs.^52,53^ Similarly, it was reported that tryptophan and tyrosine possessed reducing and capping abilities to synthesize AgNPs.^54^ Under alkaline conditions, the indole group of tryptophan and the phenolic group of tyrosine reduced Ag^+^ to AgNPs, while the oxidized tryptophan and tyrosine bound on AgNPs surface to stabilize AgNPs, which was in agreement with Joshi *et al*., who described that indole and carboxyl groups were responsible for binding tryptophan on AuNPs surface.^55^ The diameter and dispersion of AuNPs could be controlled by varying the tryptophan concentration and temperature.^56^

Beyond amino acids, nucleotides, as the basic building block of nucleic acids, can also be applied for NPs synthesis because of their H-bonding capabilities and abundant metal-binding sites. Kunoh *et al*. successfully synthesized spherical AuNPs of 5 nm utilizing RNA from iron-oxidizing bacteria and investigated the mechanism for AuNPs formation by different nucleoside parts of RNA, including guanosine, adenosine, cytidine, and uridine.^57^ The outcomes showed that guanosine exhibited the most vigorous ability to reduce Au(III) and form AuNPs, followed by adenosine, while no changes were observed in cytidine and uridine. To go further, they found the structural transformation from C–H to C–OH at guanine C8 was responsible for AuNPs synthesis.^57^

To sum up, various biomolecules have satisfactory reductive and capping ability, providing a protocol for the bioinspired synthesis of NPs. Besides, the single component and well-understood structure render biomolecules popular in mechanism research. It is of great importance to examine the mechanism for the capping of biomolecules on the NPs surface at various conditions.

### Agrowastes Recycling for BioNPs Biosynthesis

Agrowastes, as byproducts of agricultural development, are a significant concern for the food processing industry and environmental protection agencies. However, these wastes are available and consist mainly of valuable compounds, such as polyphenols, polysaccharides, proteins, alkaloids, and phenolic acids. Herein, recycling agrowastes can provide a promising protocol for NPs synthesis and lessen waste pollution severity.

Sebastian *et al*. fabricated iron oxide NPs using coconut husk extract at room temperature.^58^ They applied GC-MS to identify the reducing and capping components from coconut husk extract. Their results showed that phenolics, such as 2-hydroxypyridine, 4-hydroxypyridine, 6-hydroxyflavone, and sugar alcohol, were mainly responsible for NPs fabrication, implying that −OH and −COOH functional groups contributed to metal ions reduction. Peanut waste shell contains a high quantity of luteolin, which transforms into quinone after oxidation, donating an electron to Ag^+^ and reducing Ag^+^ to AgNPs.^59^ Krishnaswamy *et al*. applied grape seed, grape skin, and grape stalk to synthesize AuNPs with sizes ranging from 20 to 25 nm.^60^ Banana peel extracts containing polyphenols and proteins could serve as the reducing and capping agents to produce high-quality ZnONPs at low temperatures.^61^ Citrus peels comprising alcoholic and phenolic compounds could be used for producing stable AgNPs^62^ and ZnONPs.^63^

Apart from fruits peel and husk, industrial wastes are another desirable candidate for NPs synthesis. Bagasse produced by the sugar cane industry can be employed to produce AgNPs. Bagasse consists mainly of glucan, xylose and other types of oligosaccharides. These compounds possess free aldehyde or ketone groups, which can interact with NO_3_^–^ and oxidized into carboxylic acids, providing electrons to Ag^+^.^64^ Spent coffee grounds produced by the coffee industry are rich in phenolic acids, such as chlorogenic acid, feruloylquinic acid, and caffeoylquinic acid, responsible for AgNPs synthesis.^65^

Besides, agro-effluent produced by the industry is a valuable resource for NPs biosynthesis. For instance, palm oil mill effluent is rich in phenolic acids and flavonoids, providing abundant hydroxyl groups for metal ions reduction. Gan *et al*. utilized palm oil mill effluent to produce the spherical AuNPs with an average size of 18.75 nm.^66^ Calderon *et al*. synthesized FeNPs employing olive mill wastewater, which contained polyphenols and organic acids, and showed that the application of olive mill wastewater could produce a more porous structure and smaller FeNPs than traditional synthetic methods.^67^ Thus, applying agrifood waste to synthesize NPs is feasible and economical, making agriculture more sustainable.

## Bioinspired NPs for Agricultural Soils

Over the past two decades, NPs have been widely applied in agricultural practices. However, chemically synthesized NPs have adverse effects on natural living organisms, posing risks to the environment.^68^ Thus, the potential nanotoxicological effects of bioNPs on soil should be identified. Besides, heavy metals are toxic contaminants that can threaten human health through food chain contamination. In such a scenario, bioNPs have been successfully applied for the remediation of contaminated soil. Therefore, this part focuses on the ecotoxicology of bioNPs on soil and heavy-metals stabilization by using bioNPs in contaminated soil.

### Ecotoxicology of BioNPs on Soil

Soil is an ultracomplicated ecosystem and a great valuable resource. Healthy soils support agricultural productivity and sustainability.^69^ NPs have been widely applied in agricultural practices to prevent crop diseases and improve crop yield for decades. However, many studies have reported that chemical synthesized NPs might have adverse effects on natural living organisms, posing risks to the environment and human health.^70−72^ Thus, the potential nanotoxicological effects of bioNPs on soil should be identified.

Biogenic AgNPs could be a soil conditioner and provide favorable media for plant growth. The AgNPs synthesized by plant leaf (*Thuja occidentalis*) extract were evaluated for their influence on soil physicochemical properties.^73^ Interestingly, the AgNPs-treated soil was obviously crystalline and had porous structure based on SEM images. Due to the high reactivity and specific surface area, AgNPs readily aggregated with soil particles and transformed soil from blocklike aggregates into smaller crystalline particles and flakes. Besides, AgNPs significantly improved the soil quality, such as the cation exchange capacity, water holding capacity, total organic carbon, available N and P, which benefit the plant growth.^73^ Noteworthy, the deviation of optimum dosage decreased the improved effect of AgNPs. Hence, the dosage of AgNPs is a critical factor for soil quality improvement and should be considered for the application.

Besides, soil bacteria, as indispensable engineers of ecological cycles, hold the plant community and maintain ecological stability. There are essential microbial types for agricultural soils, in the order of importance, including *Actinobacteria*, *Proteobacteria*, *Acidobacteria*, *Verrucomicrobia*, *Firmicutes*, *Bacteroidetes*, *Gemmatimonadetes*, *Nitrospira*, *Chloroflflexi*, and *Planctomycetes*.^74^ They are positively associated with agricultural soil quality and crops yield. Thus, it is imperative to understand the potential impact of NPs on the soil microbial community and their functional features. Mishra *et al*. biosynthesized AgNPs using *Stenotrophomonas* sp. and found their positive influences on soil bacterial community structure and functions.^75^ They applied the qPCR method to quantify the relative abundance of soil bacterial phyla, such as *Alphaproteobacteria*, *Betaproteobacteria*, *Actinobacteria*, and *Bacteroidetes* bacterial phyla. These bacterial phyla are ubiquitous and abundant in most soil types, playing significant roles in carbon and nitrogen cycling, polysaccharide degradation, and organic matter decomposition. Their results showed that except for the relative abundance of *Bacteroidetes* group that decreased, the other three groups increased after AgNPs treatment (100 mg/kg soil), suggesting that bacterial-mediated AgNPs posed less toxicity or even benefited soil bacteria at high dosage. It is understood that the dosage and size of AgNPs, exposure time, and environmental conditions determine the AgNPs toxicity. Beyond these, the synthetic method of AgNPs (chemical or biological) also plays a vital role in AgNPs toxicity. Furthermore, the qPCR method was used to quantify bacterial functional genes for comprehending the effect of AgNPs on the bacterial nitrogen and phosphorus cycles, showing that the *NirK* and *NirS* functional genes were responsible for encoding the Cu containing nitrite reductase and cytochrome cd1 containing nitrite reductase, while the *PhoD* gene was involved in phosphorus cycling.^75^ Their results showed that AgNPs negatively impacted the relative abundance of the *NirS* gene, whereas the *NirK* and *PhoD* gene maintained unaffected. Although the effects of biosynthetic AgNPs on soil bacterial community structure and functions are well studied, the nanotoxicity behavior of bioNPs, affected by different NPs properties, such as size, shape, and different reductant types for synthesis as well as soil conditions, such as pH, organic matter content, water holding capacity, remains to be investigated.

Another impressive result was obtained by Lin *et al*., who showed that various types of beneficial bacteria increased after plant-derived Fe_2_O_3_NPs treatment.^76^ Most types of *Proteobacteria* possess Fe-oxidizing ability. The increment of *Proteobacteria* abundance might be attributed to Fe_2_O_3_NPs addition in the soil, promoting Fe(II) oxidation. *Saccharibacteria* can transform plant-derived carbon into acetate and lactate. The plant-derived biomolecules on Fe_2_O_3_NPs might contribute to the increased abundance of *Saccharibacteria*. *Acidobacteria* can oxidize Fe(II) and reduce Fe(III), and *Betaproteobacteria* identified as Fe oxidizers, both of which increased after Fe_2_O_3_NPs treatment, indicating that the redox reactions in the soil system were enhanced. Nevertheless, not all bioNPs materials have a positive effect on soil microbial growth. Ottoni *et al*. developed AgNPs using fungal (*Aspergillus tubingensis*) biomass and assessed their impact on the aerobic heterotrophs soil microorganisms by measuring the CO_2_ content released from microorganism respiration.^77^ Their results showed that the percentage of CO_2_ after the AgNPs treatment decreased by half compared with control, suggesting that the AgNPs had a substantial inhibitory effect on the aerobic heterotrophs soil microorganisms. The reasons for the different performance of AgNPs may be due to the differences in soil types, microbial community structure, or other environmental factors. Apart from these, the most crucial factors could be the difference in bioresources applied for NPs synthesis. *Aspergillus* sp. is a well-known fungus in the agricultural and food industry and is famous for the production of mycotoxins, causing severe damage to bacteria, insects, and mammals at a very low dosage.^78^ In this regard, it is of the essence to evaluate the candidates utilized for NPs synthesis and the roles of reductants from these candidates in the toxicity of bioNPs need to be elaborated.

### Remediation of Contaminated soil

With industrial developments, various toxic heavy metals can be discharged into the soils and adversely impact the soil properties and food security. As a well-known toxic contaminant in soils, hexavalent chromium (Cr(VI)) can migrate into the roots of crops and menace human health *via* the food chain. As catalytic or chemical approaches can achieve Cr(VI) removal, Wang *et al*. employed biosynthetic PdNPs utilizing *Shewanella loihica* as a powerful catalyst for reducing Cr(VI) with formic acid and attained the total removal of Cr(VI) within 3 h.^79^ This catalytic rate of *Shewanella loihica*-derived PdNPs was much faster than that of PdNPs synthesized by *Enterococcus faecalis*.^80^ The different performance of PdNPs could be attributed to the functional protein from different bacteria and the different particle sizes. To go further, the formic acid could generate hydrogen by dehydrogenating to reduce Cr(VI) as described by the equations below:
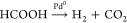
1
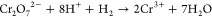
2

In such a scenario, Wang *et al*. employed hydrogen gas as a reductant instead of formic acid to reduce Cr(VI) in the presence of PdNPs. Nevertheless, a much lower reduction rate of Cr(VI) was achieved, implying that the hydrogen generated from formic acid on the surface of PdNPs might be the critical step for Cr(VI) reduction.^79^ In general, the process of Cr(VI) catalytic reduction can be proposed as that formic acid is catalytically dehydrogenated in the presence of PdNPs, the hydrogen adsorbed on PdNPs surface, and these Pd-adsorbed hydrogens then reduce Cr(VI) to Cr(III).

**Figure 3:**
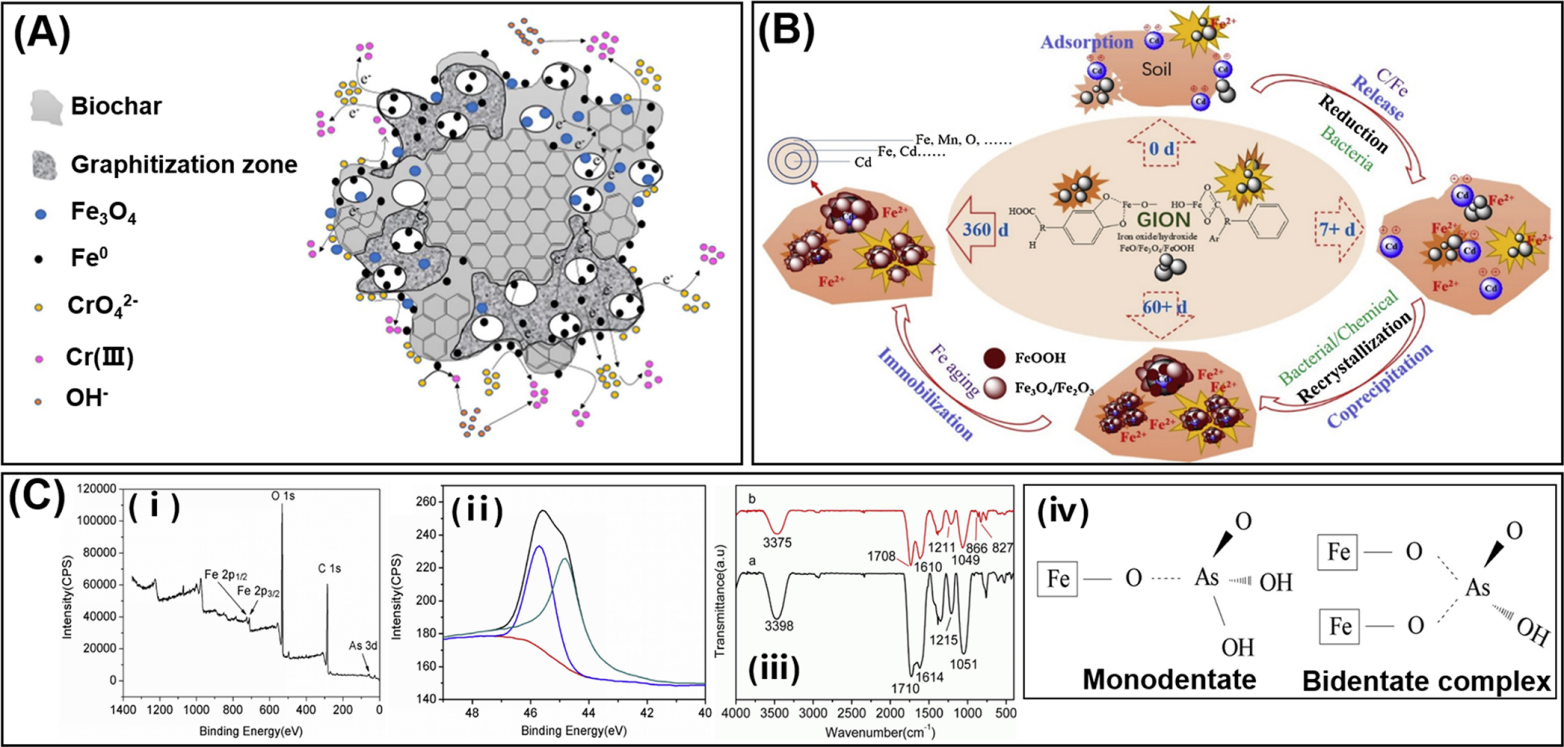
Schematic representation of bioNPs-based strategy for Cr, Cd, and As removal in soil. (A) The reaction pathway of Cr(VI) removal by biochar containing FeNPs. Reproduced with permission from ref (84). Copyright 2020 Elsevier. (B) The mechanism for Cd stabilization by green synthesized iron oxide nanoparticles (GION) in soil. Reproduced with permission from ref (76). Copyright 2019 Elsevier. (C) (i) XPS full scan spectrum of FeNPs. (ii) The As 3d narrow scan spectrum of FeNPs. Two peaks at 45.7 and 44.8 eV represented HAsO_4_^2–^ and AsO_4_^3–^, suggesting that As was adsorbed on FeNPs as As(V) without valence state variation. (iii) FTIR spectra of FeNPs before (a) and after (b) As(V) adsorption, the peak at 827 and 866 cm^–1^ represented the formation of an As–O–Fe bond in monodentate (FeO)AsO^3–^ and the stretching vibration of As–O in bidentate complex (FeO)_2_AsO^2–^. (iv) Possible complex structures of the monodentate and bidentate complex. Reproduced with permission from ref (89). Copyright 2019 Elsevier.

Regarding the chemical method of Cr(VI) removal, iron NPs possess a high reducing activity and become a prospective remediation material for the treatment of Cr(VI) contaminated soils. The conventional iron NPs production method applies borohydride as a reductant, but this chemical method is expensive and generates toxic byproducts, impeding the large-scale application. Other drawbacks like instability, agglomeration, and corrosion also restrict the development of iron NPs. On the contrary, the bioinspired synthesis is cost-effective and ecofriendly, becoming a better substitute for traditional synthesis. Luo *et al*. synthesized FeNPs by utilizing grape leaf aqueous extract and achieved 94.5% Cr(VI) removal efficiency by FeNPs under the optimum condition.^81^ This synthesized method was facile, quick, and nontoxic, providing a promising route for FeNPs production and Cr(VI) remediation. Chatterjee *et al*. reported that the superparamagnetic Fe_3_O_4_NPs prepared by the fungal biomass from *Aspergillus niger* could effectively reduce Cr(VI).^82^ The Fe_3_O_4_NPs exhibited >99% removal of Cr(VI) within 2 h at the dosage of 2.5 g/L, while the Fe_3_O_4_/activated carbon nanocomposite prepared by chemical synthesis showed 95% removal within 24 h at the dosage of 5 g/L,^83^ indicating that the performance of biosynthetic Fe_3_O_4_NPs was more superior to that of chemically synthesized Fe_3_O_4_NPs. However, the initial Cr(VI) concentration significantly impacts the Cr(VI) removal capacity upon iron NPs. When the initial Cr(VI) concentration was increased from 10 to 50 mg/L, the removal capacity was decreased from 96 to 4%.^82^ Notable progress was achieved by Liu *et al*., who employed rice husk as a reductant and carrier to produce biochar containing FeNPs (b-FeNPs) under pyrolytic reaction.^84^ The b-FeNPs could eliminate Cr(VI) in the soil leachate at an initial Cr(VI) concentration of 60 mg/L under the optimum conditions. Besides, the removal capacity of b-FeNPs was 180.85 mg-Cr/g-Fe, which was greater than those (56.6,^85^ 132.8^86^ mg-Cr/g-Fe) of biochar with FeNPs produced by the chemical method. The mechanism of Cr(VI) removal is attributed to an interaction between the surface functional groups of biochar and Cr(VI) as well as the redox reaction between FeNPs and Cr(VI). The main redox products include FeOOH, FeCr_2_O_4_, Cr(OH)_3_, and Cr_2_O_3_. First of all, the CrO_4_^2–^ combines with functional groups of biochar and then is gradually reduced to Cr(III) by FeNPs through electrons transfer from Fe^0^ to CrO_4_^2–^. The Fe^0^ is oxidized to Fe_3_O_4_ and FeCr_2_O_4_. Meanwhile, OH^–^ generated by the consumption of H^+^ in the aqueous system results in the formation of FeOOH and Cr(OH)_3_, which further converts to Cr_2_O_3_ partially (Figure 3A).

Cadmium (Cd), like other toxic heavy metals in soils, hampers plant growth and imposes several threats to humans and animals. Biogenic iron oxide NPs can stabilize Cd in contaminated soils, and the mechanism was investigated by Lin *et al*., who employed *Excoecaria cochinchinensis* leaf extract to prepare Fe_2_O_3_NPs and proposed a desorption-coprecipitation-ripening-stabilization mechanism.^76^ At first, Cd was desorbed from the soil particles and attached to the Fe_2_O_3_NPs surface. Then the Cd and Fe^2+^ on the Fe_2_O_3_NPs surface were coprecipitated by oxidation to form a Fe–Cd complex. Subsequently, excessive Fe^2+^ would trigger the recrystallization of Fe minerals on the Fe–Cd complex. Eventually, Cd was stabilized by covering the Fe–Cd complex with Fe minerals layer by layer to form multilayer and stable complexes (Figure 3B).

Arsenic (As) is a substantially toxic pollutant in soil and water, causing severe detrimental health effects *via* food chain contamination. As(V) is a prime valent state of inorganic As in the soil. Several scientists have shown that biosynthetic FeNPs and iron oxide NPs can strongly interact with As for remediation in As contaminated soils. Martínez-Cabanas *et al*. utilized eucalyptus leaves extract as a reductant to prepare iron oxide NPs, which were further encapsulated into chitosan to develop a magnetic hybrid material and reported that this magnetic hybrid material showed a capability to adsorb As.^87^ Lopez-Garcia *et al*. illustrated that pH affected the metal speciation and the surface ionization state, determining the adsorption ability of the material and showed that the As(V) adsorption capacity by hybrid material was decreased with pH increments from 7 to 11, indicating that near-neutral environment awarded the maximum adsorption capacity of hybrid material to remove As.^88^ However, the adsorption mechanism is still unknown. In the last two years, Wu *et al*. proposed the As(V) removal mechanism by biogenic FeNPs.^89^ They synthesized FeNPs by eucalyptus leaves extract, which exhibited high As(V) adsorption capacity. Based on the FTIR and XPS results, they reported that the chemical adsorption mechanism could be deduced that As(V) combined with the FeNPs surface *via* Fe–O–As bonds to form monodentate chelating ligands and bidentate binuclear complexes (Figure 3C). With respect to the contaminated soils, Su *et al*. investigated that the effect of biogenic iron oxide NPs on the distribution and transformation of As species in contaminated soils.^90^ Their outcomes showed that the biogenic iron oxide NPs could effectively stabilize As through electrostatic attraction rather than redox reaction. The surface of iron oxides is positively charged, which can electrostatically adsorb both arsenate and arsenite. As mentioned before, the Fe^2+^ generated from iron oxide NPs induces recrystallization on the surface of As–Fe complexes, and the OH^–^ released by the consumption of protons causes the coprecipitation and stabilization of As.

Overall, bioNPs provide a low-cost and ecofriendly approach for the remediation of heavy-metal contaminated soils. Although the exceptional performances of bioNPs on heavy-metal remediation have well been studied, the ameliorative mechanism and roles of the biomolecules involved NPs synthesis in heavy-metal remediation need to be further studied in the future for their better applications.

## Bioinspired NPs for Crop Growth

With enormous progress in nanotechnology, the application scale of NPs has been steadily expanding over the years. It raises concern that the concentration of NPs will increase in soil, and the accumulation of NPs poses potential threats to the environment and food safety. Fortunately, bioinspired synthesis can render NPs more stable and biocompatible by modifying the surface of bioNPs with natural substances. Several investigations revealed that bioNPs could improve seedling growth and mitigate the phytotoxicity of crops. This section aims to discuss how the bioNPs influence seed germination, seedling growth, and physiological performance of the crop and evaluate the phytotoxicity of bioNPs compared to chemically synthesized NPs.

### Seed Germination and Seedling Growth

**Figure 4:**
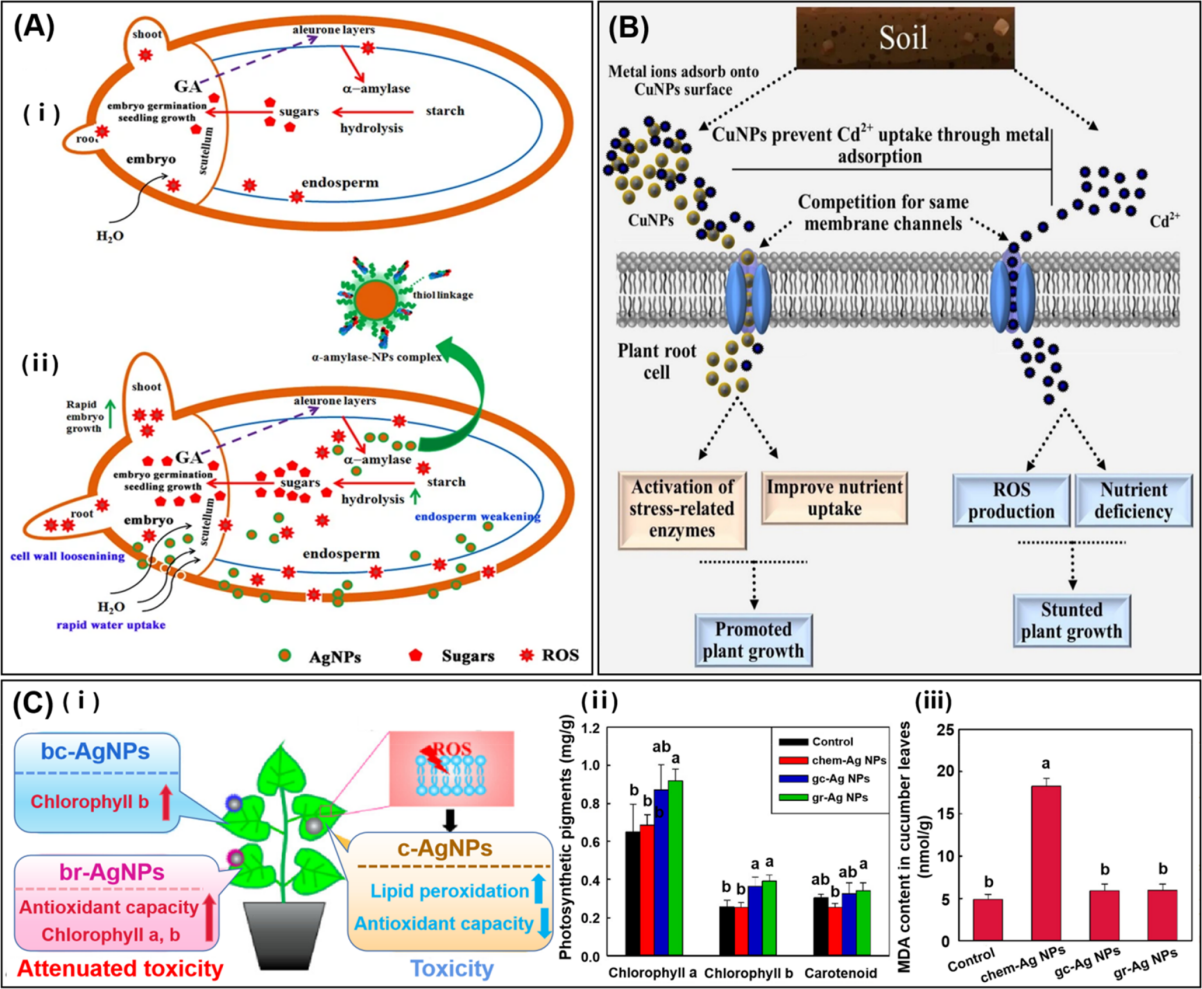
Effect of bioNPs on seed germination, heavy-metal stress, and toxicity of plant. (A) Proposed mechanism of AgNPs induced seed germination. (i) Seed without AgNPs priming. (ii) Seed with AgNPs priming, which facilitated seed germination through three possible routes. First, the AgNPs created small pores in cell walls leading to higher water uptake. Second, the penetrated AgNPs interacted with α-amylase as α-amylase-NPs complex to promote starch hydrolysis, producing more sugars to support embryo growth. Third, the AgNPs mediated the generation of ROS to loosen the cell wall and weaken the endosperm. Reprinted with permission under a Creative Commons Attribution 4.0 International License from ref (92). Copyright 2017 Springer Nature. (B) Proposed mechanism for the inhibitory effect of CuNPs on the Cd translocation from soil to plant cell. Reproduced with permission from ref (109). Copyright 2020 Elsevier. (C) (i) The toxicity of b-AgNPs is less than that of c-AgNPs. (ii) The photosynthetic pigments and (iii) the MDA contents of cucumber leaves for four treatments: control, chem-Ag NPs, gc-Ag NPs, and gr-Ag NPs. Reproduced with permission from ref (125). Copyright 2021 Elsevier.

Rapid, uniform, and successful seed germination and seedling growth are crucial phases of crops for agricultural production. Seed priming is a prospective strategy to ensure a high germination rate, which hydrates seeds with a certain priming solution. The commonly used priming solutions are water, inorganic salts, polyethene glycol, and hormone.^91^ In recent years, bioNPs as priming agents have proven to be more effective and efficient than conventional priming solutions. Mahakham *et al*. used *Citrus hystrix* D.C. leaf extract to prepare the AgNPs and investigated the mechanism regarding the positive effect of biosynthetic AgNPs priming on aged rice seeds germination (Figure 4A).^92^ Their outcomes presented that priming with AgNPs solution distinctly promoted the seedling biomass and water uptake compared with hydropriming. A sufficient amount of water is a prerequisite to initiating cellular metabolism and growth. Thus, the process of water uptake plays a vital role in seed germination. To go further, they also found that aquaporin genes, including *PIP1;1* and *PIP2;1*, were upregulated. As a transmembrane protein, aquaporins are responsible for transporting water, nutrients, and CO_2_ into a cell. Besides, the contents of α-amylase and soluble sugar were elevated by the AgNPs treatment, suggesting that the AgNPs could enhance the starch metabolism of rice seedlings. The α-amylase belongs to a hydrolytic enzyme, contributing to the decomposition of polysaccharides into monosaccharides, supplying energy for respiratory metabolism and cell growth. Thus, the faster germination rate in AgNPs-treated seed could be partially explained by the increased content of α-amylase. Meanwhile, AgNPs priming seed produced greater reactive oxygen species (ROS) levels and enhanced antioxidant enzymes activities than hydropriming.^92^

A recent concept has been proposed that germination requires a certain amount of ROS defined as the oxidative window. Out of the oxidative window, germination does not occur. As ROS perform as a signal molecule, they facilitate seed germination and seedling growth. However, excessive ROS accumulation causes damage to the cell and the need for regulation by antioxidant enzymes. Several studies reported that a primed seed showed more superior antioxidant properties than an unprimed one under adverse conditions.^93,94^ In this context, studying how antioxidant enzymes balance ROS within the oxidative window for seed germination is of great importance. However, the precise mechanism is not well understood, and more works are needed in the future.

Along with AgNPs, Acharya *et al*. produced AuNPs using onion extract and investigated their influences on the growth and metabolomics of onion seed.^95,96^ AuNPs priming effectively improved the yield and quality of onion. The peroxidase (POD) as a primary antioxidant enzyme increased significantly after AuNPs treatment. Their TEM images presented that the AuNPs accumulated in the seeds cells after AuNPs priming, substantiating that the AuNPs penetrated the seed cell. Primed seeds imbibe water and produce ROS. These ROS cause the loosening of the cell wall to stimulate germination and the AuNPs might mediate the production of ROS in the oxidative window to enhance seed germination. Besides, the content of germination inhibitors, such as jasmonic acid (JA), 12-oxophytodienoic acid (OPDA), and abscisic acid (ABA), decrease in a AuNPs-treated onion, whereas the content of germination stimulators, such as zeatin (ZA) andγ-aminobutyric acid (GABA), increased. Another research reported that plant-derived iron oxide NPs decreased OPDA levels in watermelon seedlings, effectively breaking seed dormancy.^97^ These alterations in the germination inhibitors and stimulators might explain the improved seed emergence after NPs priming. Therefore, bioNPs as a priming agent have great potential to improve seed performance and seedling growth.

Other than applications in seed priming, AgNPs can directly impregnate into a medium to support rice growth. Gupta *et al*. investigated the effect of plant-mediated AgNPs on the growth of rice seeds, in which the seeds were germinated on an agar medium supplemented with AgNPs in a flask, and found that the AgNPs synthesized from rhizome extract of *C. orchiodes* could boost the shoot and root growth of the rice seedlings.^98^ To understand the mechanism of the improvement, they focused on analyzing the ROS content, antioxidative enzyme activity, and related gene expression level in rice leaves with AgNPs treatment, and showed that the malondialdehyde (MDA) and H_2_O_2_ content decreased while the catalase (CAT), ascorbate peroxidase (APX) activities increased and the related genes expressions were upregulated. These antioxidative enzymes are responsible for the ROS quenching, mitigating the severity of ROS damage in the course of seedling growth, and the plant-derived AgNPs facilitated the seedling growth by stimulating the efficient ROS defense mechanism and enhancing the activities of the antioxidative enzymes to reduce the ROS level. Apart from CAT and APX, superoxide dismutase (SOD) and POD also reduce ROS accumulation in plants. Interestingly, Gupta *et al*. also found that the SOD activity increased, whereas the *CuZnSOD* gene was downregulated after exposure to AgNPs, indicating that this enzyme activity might not be affected by mRNA levels but posttranscriptional level.^98^ The AgNPs developed from *Tagetes erecta* (marigold) leaf and flower extracts also enhanced maize plant growth by spraying on the plant after seeds sowing.^99^

Due to the low solubility in soils, native phosphorus (P) utilization by crops is relatively inefficient, restricting the growth and productivity of the crops. Raliya *et al*. applied biosynthetic ZnONPs to improve the P availability for plants. The ZnONPs were synthesized using the cell-free filtrate of soil fungus *Aspergillus fumigatus* TFR-8 and results showed the enhancement of the native P uptake by the mung bean and cluster bean.^100,101^ Zinc serves as a cofactor for phosphatase and phytase, which hydrolyze the ester bonds between P and other metal elements (Fe, Al, or Ca), rendering the native P more available for the plant roots.^102^ ZnONPs stimulate these P, mobilizing enzymes activity and facilitating plant metabolism. Besides, zinc is a necessary micronutrient in plants, which benefits seed germination and seedling growth.^103^ ZnONPs prepared by plant leaf extract (*Aloe barbadensis Mill*) could act as a nutrient source for wheat seed growth.^104^ The shoot and root lengths of wheat seeds were increased after plant-derived ZnONPs treatment compared with chemical-derived ZnONPs treatment. On the one hand, this could be attributed to the smaller size of biological ZnONPs compared with chemical ZnONPs, resulting in a greater zinc uptake and wheat seedling growth.^105^ On the other hand, the plant leaf extract applied for NPs synthesis contains many active components, such as tannins, flavonoids, and polyphenols, on the ZnONPs surface, facilitating the shoot and root growth.^106^ Nevertheless, several investigators reported that zinc in excess could increase ROS production detrimental to plant growth. The ZnONPs synthesized from the flower extract of *Elaeagnus angustifolia* illustrated affirmative influences on tomato seeds at a lower concentration, while higher ZnONPs concentrations were detrimental to seed germination and growth.^107^ The α-amylase-mediated ZnONPs improved *Brassica juncea* seed germination at the dosage of 20 μg/mL, whereas higher ZnONPs concentration significantly decreased the seed germination.^94^ As such, it is essential to optimize the concentration of NPs for precise application.

### Heavy-Metal Stress

Typical heavy metals, such as chromium (Cr) and cadmium (Cd), are found in soils, limiting agricultural yield and development. The uptake of Cr(VI) by plants causes adverse impacts on plant growth, including the variations of physiological processes and the production of ROS. Fortunately, NPs synthesized by bioinspired approaches can overcome these problems. The CuNPs, which were synthesized by the *Klebsiella pneumoniae* strain, could effectively mitigate the toxicity of Cr(VI)-contaminated soils to wheat plants.^108^ The root length and shoot length of CuNPs-treated wheat plants increased significantly under the Cr stress compared with the plants without CuNPs treatment. The elevating cellular antioxidants such as catalase, peroxidase, proline, and phenolic compounds in CuNPs-treated plants were obtained. Thus, this ameliorative mechanism might be attributed to the stimulating effect of bacterial-derived CuNPs on cellular antioxidant activities in wheat plants, alleviating the ROS damage to plant cells. Besides, the Cr translocation and accumulation in root and shoot were minimal after supplying bacterial-derived CuNPs to the soil at the dosage of 50 mg/kg soil, but the mechanism remains elucidated.^108^ Another research from the same authors provided the mechanism associated with the inhibitory effect of bacterial-derived CuNPs on the Cd translocation from soil to wheat plants.^109^ This mechanism might also explain the ameliorative effect of CuNPs on Cr translocation and accumulation in wheat plants (Figure 4B). On the one hand, the larger specific surface area and high reactivity of CuNPs contribute to the immobilization of Cd onto the CuNPs through electrostatic attraction. This immobilization is supported by the increased content of residual Cd in the postharvest soil. On the other hand, the competition between CuNPs and Cd at transport sites of root cells occurs. This competition is evidenced by the increased level of Cu in plant roots and shoots. Furthermore, elevating nutrient contents such as N and P in CuNPs-treated wheat plants were achieved, and Cu as an enzyme cofactor activated various crucial enzymes associated with plant growth.^109^ In this context, biosynthetic CuNPs can perform as robust remediation material to mitigate the toxicity and facilitate plant growth under the Cr or Cd stress.

It has been widely investigated that Cd reduces chlorophyll content and photosynthetic activity, changes enzymatic activities, and retards plant growth and yield.^110,111^ Sebastian *et al*. synthesized iron oxide NPs by using coconut husk extract rich in phenolics and reported that these iron oxide NPs ameliorated Cd stress and fueled Fe in rice plants, improving the biomass, chlorophyll content, and quantum yield of photosynthesis in rice plants.^58^ Fe is an essential element for respiration, photosynthesis, and chlorophyll biosynthesis.^112^ In this regard, the phenolics-derived iron oxide NPs can act as a Cd remover and Fe fertilizer to promote agricultural yield under heavy-metal stress and Fe deficiency. However, the maximum adsorption capacity (MAC) of as-developed iron oxide NPs for Cd was 9.6 mg/g, which was comparatively lower than chemically synthesized iron oxide NPs with MAC of 19.59,^113^ 27.83,^114^ 45.66^115^ mg/g. The variations in the parameters, such as pH, temperature, and metal ion concentration, could contribute to the variations in the metal adsorption by the NPs.^116^ More importantly, the MAC of magnetite NPs relies on synthetic method and fabricated material property responsible for the structure, functional groups and surface area of NPs. These features affect the number of available and active sites in NPs, determining the total amount of metal ions adsorbed onto the NPs surface.^117^ Although the bioinspired synthesis of iron oxide NPs is cost-effective and ecofriendly, the low MAC means a high cost in applications, hindering the applicability of NPs in heavy-metal removal. Sebastian *et al*. achieved notable progress after a year, reaching the MAC of 37.03 mg/g in using *Hevea* bark extract to synthesize the magnetite NPs.^118^ The rice growth was inhibited in the Cd-spiked soil, whereas the ameliorative situation was found by magnetite NPs amendment. Accumulation of heavy metal causes ROS production, destroying the membrane in plant cells and increasing the MDA content.^119^ Sebastian *et al*. also discovered that the MDA content decreased, while peroxidase, which is synthesized by cell and responsible for the radicals scavenging, was unaffected after NPs treatment.^118^ These outcomes suggested that magnetite NPs could alleviate the ROS damage by minimizing Cd accumulation rather than stimulating the antioxidative defense mechanisms of plants. However, opposite results were obtained by one other similar research, in which the iron oxide NPs were synthesized by utilizing the supernatant of bacterial strain (*Pantoea ananatis*), the growth of wheat plants was enhanced under Cd stress by adsorption of Cd on the NPs surface, and the concentrations of SOD and POD were increased in the plant after iron oxide NPs treatment.^120^ The ROS production induces enzymatic defense mechanisms in the plant, maximizing SOD and POD to reduce the detrimental impacts of lipid peroxidation products.^119^ Thus, biosynthetic iron oxide NPs can stimulate plant growth under Cd stress, which is partially attributed to the elevation of antioxidant enzymes. The variation in POD concentration in NPs-treated plants can be ascribed to the difference in plant species and synthetic materials, for example, plant extract and bacterial supernatant. Nevertheless, the roles of synthetic material in NPs for plant growth under heavy-metal stress are still unexplored, and further research is needed. Generally speaking, the chemical synthetic process of NPs requires more economical and labor input as well as toxic reagents, compared with the bioinspired synthetic procedure. Therefore, the biogenic NPs could be a valuable resource to improve yield under heavy-metal stress for safe and sustainable agricultural practice.

### Phytotoxicity

As mentioned previously, the accumulation of NPs in soils poses potential threats to the environment and food safety, and studies have reported that NPs at high concentrations in soils negatively impact crop yield and quality.^121−124^ Fortunately, bioinspired synthesis can render NPs more stable and biocompatible by modifying the surface of bioNPs with natural substances. The bioNPs can not only improve seedling growth and quality of crop but also mitigate the phytotoxicity of crop. Zhang *et al*. developed biosynthetic AgNPs (b-AgNPs) using rice husk extracts and cucumber leaves and employed cucumber as a model plant to evaluate the phytotoxicity of b-AgNPs compared with chemical AgNPs (c-AgNPs) (Figure 4C(i)).^125^ Their results showed that the b-AgNPs substantially enhanced cucumber plant photosynthesis by increasing chlorophyll contents (Figure 4C(ii)). As the end products of lipid peroxidation, MDA contents reflect the extent of lipid peroxidative damage on the cell. When cucumber was exposed to c-AgNPs, the MDA content elevated 3.7 folds compared with control, whereas the MDA content remained unchanged after exposure to b-AgNPs, indicating that the toxicity of b-AgNPs was less than that of c-AgNPs (Figure 4C(iii)). In agreement with Kannaujia *et al*., the b-AgNPs prepared by fruit extract of *Phyllanthus emblica* L. and assessed based on their phytotoxicity in terms of ROS production assay in wheat plants compared with c-AgNPs revealed that the increment in ROS accumulation in wheat plants was in the order of c-AgNPs > c-AgNPs + fruit extract > b-AgNPs.^126^ These results demonstrated that the fruit extract abundant in antioxidants possesses ROS scavenging capacity and b-AgNPs equipped with these plant-derived antioxidants become more biocompatible and less phytotoxic. Besides, the Ag bioaccumulation in b-AgNPs treated wheat plants was substantially lower than that of the c-AgNPs treated one.^126^ As Ag^+^ ions can easily interact with proteins and inactivate enzymes, the reoxidation from Ag^0^ to Ag^+^ ions can thus account for the toxic effects of AgNPs in a plant. The c-AgNPs are vulnerable to oxidation, whereas the biogenic antioxidants capping on the b-AgNPs surface award the b-AgNPs more stable and less phytotoxic.

Besides, the phytotoxicity of the AgNPs varies with their concentrations and plant species. *Ferula persica* leaf extract was employed to produce b-AgNPs, and high concentrations of b-AgNPs ranging from 100 to 600 ppm were applied in basil seeds to analyze the toxicity, showing that the applications of b-AgNPs and c-AgNPs negatively impacted shoot and root elongation at high concentrations.^127^ The maximum shoot (29.5 mm) and root (44.75 mm) lengths were achieved without any treatment. After 600 ppm b-AgNPs treatment, the minimum values of 21.63 mm in the shoot and 16.25 mm in the root were observed, while 400 ppm c-AgNPs completely inhibited the shoot and root elongation. These results suggested that b-AgNPs exhibited lower phytotoxic effects on basil seed growth than c-AgNPs. Furthermore, investigating b-AgNPs at low concentration ranges is of significance for phytotoxic analysis and precise application. Kim *et al*. prepared b-AgNPs by adopting *Laminaria japonica* algal extract, and the seedling growth of wheat was analyzed with low concentrations of AgNPs from 10 to 80 ppm, showing that the growth of wheat seed remained unchanged after exposure to 30 ppm b-AgNPs, whereas the growth decreased significantly after exposure to b-AgNPs at a higher level of 40 ppm, providing a guideline that applying b-AgNPs at a dose of 30 ppm was appropriate for commercial applications.^128^ With respect to c-AgNPs, the decrement of wheat growth was observed when exposed to 20 ppm c-AgNPs.^128^

Although the attractive benefits of bioNPs in biomedicine^20,129^ and catalyst^130,131^ are obvious, there is still limited research available for agricultural applications. BioNPs have leading-edge attributes compared with chemically developed NPs. For instance, they possess substantial biocompatibility and minor toxicity for crops. Also, they are stable biological materials during application and storage without apparent ion release. The synthesis has a low cost and is facile without involving harmful reagents. These favorable traits have attracted interest to explore their applications for agricultural purposes. However, the biological materials applied for synthesis are complex and not all these materials can award NPs a positive performance. Therefore, the mechanisms regarding the roles of fabricated materials in the applications of bioNPs remain to elucidate, and exploring more effective fabricated materials is still needed.

## Bioinspired NPs for Crop Disease Managements

Crop diseases and pest attacks are primarily responsible for the destruction of crops and loss of agricultural yield. This section aims to introduce common crop diseases caused by a fungal or bacterial pathogen and discusses current advances in bioNPs against phytopathogens and pests compared to chemical NPs.

### Fungal Disease Management

**Figure 5:**
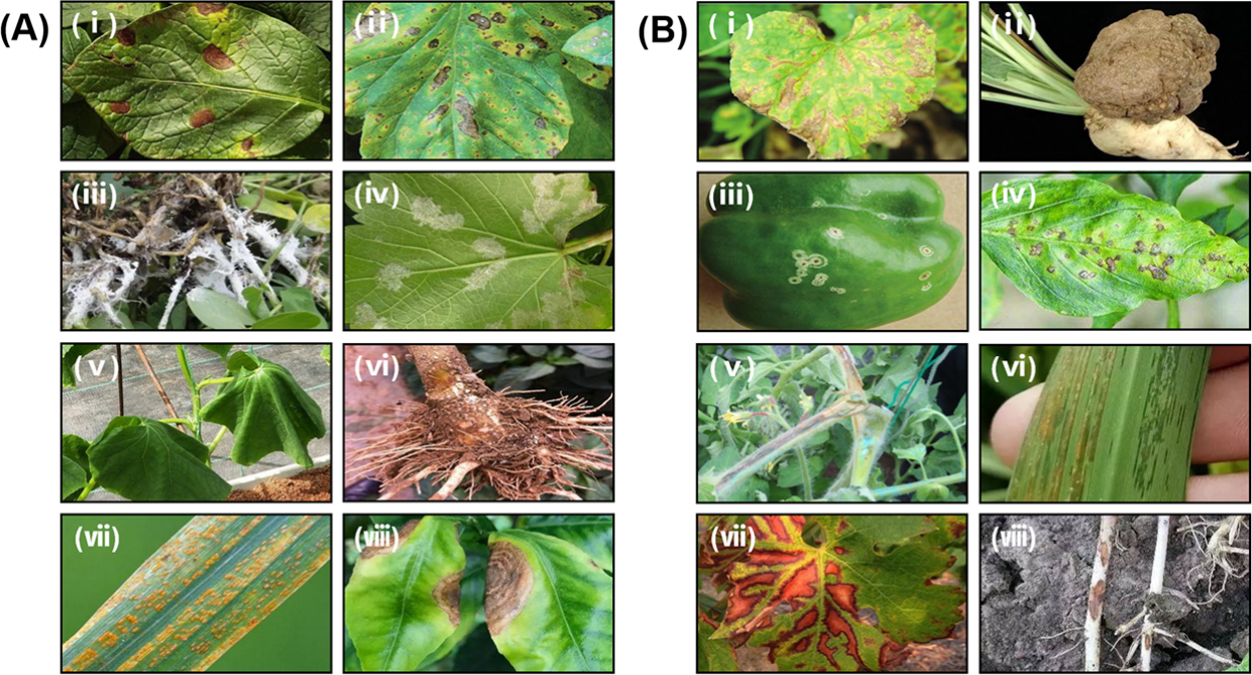
Various plants showing symptoms of common fungal or bacterial plant disease. (A) Various symptoms of fungal plant disease: (i) Leaf blight of potato, (ii) leaf spot of tomato, (iii) white mold of peanut, (iv) downy mildew of grape, (v) vascular wilt of cucumber (vi) root rot of pepper, (vii) wheat rust, and (viii) anthracnose of citrus. (B) Various symptoms of bacterial plant disease: (i) Bacterial leaf wilt of muskmelon, (ii) crown gall disease of radish, (iii) bacterial canker disease of pepper, (iv) bacterial spot of pepper, (v) bacterial wilt of tomato, (vi) bacterial brown strip disease of rice, (vii) Pierce’s disease of grape, and (viii) blackleg disease of potato.

Crop diseases caused by fungal pathogens occur at a particular stage of plant growth, confining agricultural economic development. Fungal plant pathogens can survive by absorbing the nutrients in the plant or destroying the plant cell to liberate the nutrients and utilize them. Major fungal plant pathogens are *Colletotrichum* sp., *Pestalotiopsis* sp., *Fusarium* sp., *Ustilago* sp., *Alternaria* sp., *Rhizoctonia* sp., *Sclerotium* sp., *Sclerotinia* sp., *Phytophthora* sp., *Botrytis* sp., *etc*. These fungi penetrate the plant by entering the stomata or attack the plant by secreting enzymes that alter the plant surface. The symptoms of plants infected by fungi appear as leaf blight, leaf spot, white mold, downy mildew, vascular wilt, root rot, *etc*. (Figure 5A).

Chemical methods like chemical pesticides, biological methods like microbial agents and genetic engineering, and their combination methods are generally applied to manage crop diseases. On the other hand, NPs are also commercially applied for pathogen management due to their excellent performance in antimicrobial activity. However, the potential toxicity and high cost of traditional NPs limit their broad applications. Alternatively, bioNPs are more compatible and cost-effective, providing a strategy to manage crop diseases. Over the past decade, considerable research has substantiated that bioNPs possess robust antifungal activity. The antimicrobial activities of bioNPs against phytopathogens are summarized in Table 3. There are several popular methods to evaluate the antifungal activity of NPs, including fungal growth in potato dextrose agar (PDA) or potato dextrose broth medium (PDB) that contains NPs. SEM and TEM can also be used to observe the morphology and ultrastructure of a microbial cell as affected by the antimicrobial activity of bioNPs.

**Table 3:** Antimicrobial Activity of BioNPs against Common Phytopathogens

s. no.	types of NPs	sources	pathogens	method	result	ref
Fungicide						
1	Ag	*Streptomyces griseoplanus* SAI-25 (fungus)	*Macrophomina phaseolina*	PDA	13 mm inhibition zone at 1000 ppm	(278)
2	Ag	*Melia azedarach* (plant)	*Verticillium dahlia*	PDA	51% growth inhibition at 60 ppm	(279)
3	Ag	*Trichoderma* sp. (fungus)	*Fusarium oxysporum* f. sp. *ciceri*.	PDA	95% growth inhibition at 100 ppm	(280)
4	Ag	*Ganoderma applanatum* (basidiomycete)	*Botrytis cinerea* and *Colletotrichum gloeosporioides*	leaflet assay	100% growth inhibition for *B. cinerea* and 100% growth inhibition for *C. gloeosporioides* at 50 ppm	(281)
5	Ag	*Bacillus sp.* AW1–2 (bacterium)	*Colletotrichum falcatum* Went	PDA	>80% growth inhibition at 20 ppm	(132)
6	Ag	rice leaf (plant)	*Rhizoctonia solani* TS-06, TS-10, TS-14, TS-20, TS-22, and TS-24	PDA	82–97% growth inhibition at 10 ppm	(144)
7	Ag, TiO_2_, and Se	*Aspergillus versicolor* (fungus)	*Alternaria alternate*	PDA	77% growth inhibition at 100 ppm Ag, 90% growth inhibition at 100 ppm TiO_2_, 90% growth inhibition at 100 ppm of Se	(282)
8	Cu	chitosan (polysaccharide)	*Rhizoctonia solani* and *Pythium aphanidermatum*	PDA	94% growth inhibition for *R. solani* and 98% growth inhibition for *P. aphanidermatum* at 1000 ppm	(283)
9	Cu	curcumin (plant)	*Fusarium oxysporum* f. sp. *ciceri*.	PDA	65% growth inhibition at 200 ppm	(284)
10	Fe_2_O_3_	*Azadirachta indica* (plant)	*Fusarium oxysporum*	PDA	88% growth inhibition at 1000 ppm	(285)
11	ZnO	Garlic (plant)	*Colletotrichum* sp. and *Mycena citricolor*	PDA	93% growth inhibition for *Colletotrichum* sp. and 97% growth inhibition for *Mycena citricolor* at 12 mmol/L	(138)
12	MgO	*Burkholderia rinojensi* (bacterium)	*Fusarium oxysporum* f. sp. *lycopersici*.	PDA	100% growth inhibition at 15.36 ppm	(136)
13	Se	*Trichoderma atroviride* (fungus)	*Phytopthora infestans*	green house	tomato seed primed by 100 ppm of SeNPs exhibited 72.9% protection against late blight disease caused by *P. infestans*	(148)
14	MgO	*Bacillus sp. RNT3 (bacterium)*	*Rhizoctonia solani*	PDA and PDB	81% growth inhibition in PDA and 74% growth inhibition in PDB at 100 ppm	(149)
Bactericide						
1	Ag	*Phyllanthus emblica* (plant)	*Acidovorax oryzae*	agar well diffusion and MIC	20 mm inhibition zone and 62.41% reduction at 30 ppm	(151)
2	Ag	*Lantana camara* L. (plant)	*Ralstonia solanacearum*	agar disc diffusion, swarming motility, and MIC	18 mm inhibition zone, 87% swarming inhibition, and 96% reduction at 10 ppm	(286)
3	Ag	*Solanum torvum* (plant)	*Xxanthomonas axonopodis* pv*punicae* and *Ralstonia solanacearum*	agar disc diffusion and MIC	11 mm inhibition zone for *X. axonopodis* and 18 mm inhibition zone for *R. solanacearum* at 50 ppm. 99% reduction for X. axonopodis at 6.25 ppm and 99% reduction for *R. solanacearum* at 12.5 ppm	(287)
4	Ag	*Hypericum perforatum* (plant)	*Ralstonia solanacearum*	agar disc diffusion and MIC	45 mm inhibition zone and 99% reduction at 30 ppm	(288)
5	Ag	*Fusarium oxysporum* (fungus)	*Xanthomonas axonopodis* pv *citri*	MIC	99% reduction at 6.55 ppm	(289)
6	Ag	*Taraxacum officinale* (plant)	*Xanthomonas axonopodis* pv *citri* and *Pseudomonas syringae*	agar disc diffusion	22 mm inhibition zone for *X. axonopodis* and 20 mm inhibition zone for *P. syringae* at 30 ppm	(290)
7	Ag	*Bacillus cereus* SZT1 (bacterium)	*Xanthomonas oryzae* pv *oryzae*	agar well diffusion and MIC	25 mm inhibition zone and 91% reduction at 20 ppm	(154)
8	Ag	pine cone (plant)	*Pseudomonas syringae* and *Xanthomonas oryzae* pv *oryzae*	MIC	99% reduction for *P. syringae* at 6 ppm and 99% reduction for *X. oryzae* at 11 ppm	(291)
9	Ag	sumac (plant)	*Pseudomonas syringae*	MIC	99% reduction for *P. syringae* at 12 ppm	(156)
10	Ag	onion (plant)	*Pseudomonas syringe* and *Erwinia* sp.	MIC	99% reduction for *P. syringae* at 90 ppm and 99% reduction for *Erwinia* sp. at 70 ppm	(292)
11	Ag	alginate (polysaccharide)	*Agrobacterium tumefaciens*	agar disc diffusion	>20 mm inhibition zone at 20 μg	(293)
12	ZnO	*Thymbra spicata* var. *spicata* L. (plant)	*Clavibacter michiganensis* subsp. *Michiganensis*, *Pseudomonas syringae* pv *Phaseolicola, Pseudomonas cichorii*, and *Pectobacterium carotovorum* subsp. *Carotovorum*	agar disc diffusion	37 mm inhibition zone for *C. michiganensis*, 22 mm inhibition zone for *P. syringae*, 22 mm inhibition zone for *P. cichorii*, and 18 mm inhibition zone for *P. caratovorum* at 10 μL	(294)
13	ZnO	green tomatoes (plant)	*Xanthomonas oryzae* pv *oryzae*	agar well diffusion and swarming motility	3 mm inhibition zone and 25% swarming inhibition at 16 ppm	(295)
14	MgO	*Bacillus* sp. RNT3 (bacterium)	*Acidovorax oryzae*	agar well diffusion, swarming motility, and MIC	34 mm inhibition zone, 77% swarming inhibition, and 89% reduction at 30 ppm	(149)
15	MgO	*Acinetobacter johnsonii* RTN1 (bacterium)	*Acidovorax oryzae*	agar disc diffusion, swarming motility, and MIC	35 mm inhibition zone, 65.38% swarming inhibition, and 81% reduction at 20 ppm	(150)

**Figure 6:**
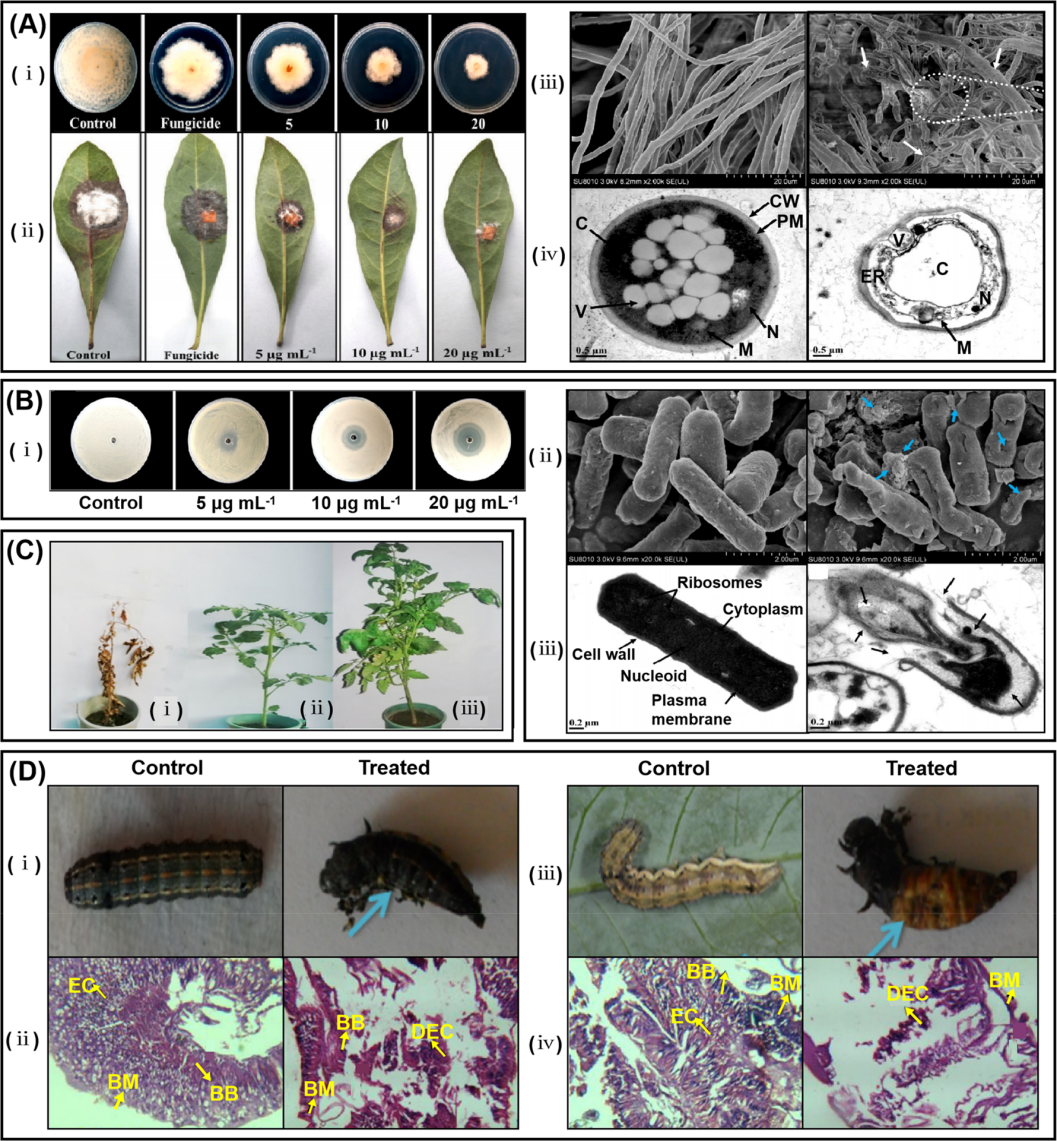
Crop disease management against fungal pathogen, bacterial pathogen, and pest by utilizing bioNPs. (A) (i) Antifungal activity of bio ZrONPs against *P. versicolor* was determined by swarming motility assay. (ii) Effect of bioZrONPs on bayberry leaves infected with *P. versicolor*. (iii) SEM and (iv) TEM microscopy images of *P. versicolor* cells treated with sterile water (left) or 20 μg mL^–1^ bioZrONPs (right). CW, cell wall; PM, plasma membrane; N, nucleus; V, vacuoles; M, mitochondrion; C, cytoplasm; ER, endoplasmic reticulum. Reproduced with permission from ref (133). Copyright 2021 Elsevier. (B) (i) Antibacterial activity of bio MgONPs against *A. oryzae* by well diffusion assay. (ii) SEM and (iii) TEM images of *A. oryzae* cells treated with sterile water (left) or 20 μg mL^–1^ bio MgONPs (right). Reproduced with permission from ref (150). Copyright 2021 Elsevier. (C) Growth of *R. solanacearumin*-challenged tomato treated with (i) sterile water, (ii) *S. laureola* leaf extract, or (iii) *S. laureola*-derived Fe_2_O_3_-NPs. Reproduced with permission from ref (152). Copyright 2019 Elsevier. (D) (i, (iii) Larvicidal activity of bio AgNPs against *S. litura* (left) and *H. armigera* (right). (ii, iv) Effect of bio AgNPs on midgut tissues of *S. litura* (left) and *H. armigera* (right). BB, brush border; BM, basement membrane; EC, epithelial cells; DEC, destructed epithelial cells. Reproduced with permission from ref (175). Copyright 2020 Springer Nature.

*Colletotrichum falcatum* Went induces red rot disease of sugar cane worldwide, posing devastating losses in the sugar industry by decreasing sugar cane yield and sugar content. Chemical fungicides like carbendazim have been applied to conquer this difficulty over the past decades. The overuse of fungicides results in fungal resistance and environmental deterioration. For sustainable agriculture production, the biosynthetic AgNPs prepared by the *Bacillus* sp. strain were evaluated for the potential antifungal feature against *C. falcatum* Went, and the results showed that the AgNPs minimized the mycelia growth compared with carbendazim, AgNO_3_, and bacterial culture supernatant at the same dosage of 20 ppm due to the size, shape, and capping proteins of AgNPs.^132^*Pestalotiopsis versicolor* is a primary pathogen causing twig blight disease in bayberry trees. Ahmed *et al*. first synthesized ZrONPs by utilizing the cultural supernatant from *Enterobacter* sp. strain and assessed their antifungal activity against *P. versicolor*, showing that the inhibitory effect of the biosynthetic ZrONPs (75%) on *P. versicolor* growth in bayberry leaves was more substantial than that of difenoconazole fungicide (20%) at the same concentration of 20 ppm (Figure 6A(i-ii)).^133^ The SEM and TEM images showed the apparent shrinkage in cell morphology and disintegration in internal organelles of *P. versicolor* cells after ZrONPs treatment (Figure 6A(iii-iv)). Besides, *Fusarium* sp. causes various diseases in crops, such as wilt, rot, blight, and canker^134^ and produces diverse mycotoxins, including fusaric acid, trichothecenes, fumonisins, and zearalenone,^135^ imposing enormous losses in agricultural and food industry. The biogenic MgO synthesized from *Burkholderia rinojensis* strain completely inhibited the growth of *Fusarium oxysporum* at the dosage of 15 ppm.^136^

An increasing number of studies have substantiated that the antifungal activity of bioNPs is more favorable than chemical NPs.^137−139^ For instance, *Ustilago tritici* belongs to a seed-borne pathogen responsible for wheat rust disease. Irshad *et al*. prepared TiO_2_NPs using plant extracts of *Chenopodium quinoa* or *Trianthema portulacastrum* or by a chemical approach and showed that the two types of plant-derived TiO_2_ presented more potent antifungal activity against *U. tritici* compared with chemical-derived TiO_2_.^140^ Besides, *Alternaria alternata* exists ubiquitously in air and soil, causing leaf spot and blight of tomatoes as well as black spot rot of apple fruits. Ali *et al*. utilized leaf extract (*Azadirachta indica)* to produce ZnONPs and the plant-synthesized ZnONPs showed more substantial inhibition against *A. alternata* than chemical-synthesized ZnONPs.^141^ Both *Alternaria* sp. and *Fusarium* sp. were more sensitive to the *Trichoderma*-modified SeNPs than traditional produced SeNPs.^142^ Furthermore, *Rhizoctonia solani* is identified as a destructive pathogen leading to the sheath blight disease of rice. The mycelia inhibition rate of 85% against *R. solani* was achieved using commercial AgNPs at the dosage of 50 ppm,^143^ while exposure to a lower concentration of plant-mediated AgNPs of 10 ppm could also achieve mycelia inhibition rates of 82–97%,^144^ which could be attributed to the biomolecular attached to the NPs, potentiating bioNPs more satisfactory antifungal activity than chemical-developed NPs. Noteworthy, after plant-mediated AgNPs treatment (10 ppm), the percent disease incidence in rice was 36–68%, which was inconsistent with the results of the mycelia inhibition rate, as pathogens can induce crop disease at low concentration of AgNPs treatment and thus higher levels of NPs are needed for controlling crop diseases.^144^

*Sclerotium rolfsii*, as a soil-borne phytopathogen, can produce sturdily constructed sclerotia to survive for a long-term under an adverse environment in soil and attack crop seedlings under favorable conditions. Therefore, controlling sclerotia germination is a primary strategy to manage the *S. rolfsii*-caused diseases. The AgNPs biosynthesized by the culture supernatant of *Stenotrophomonas* sp. were substantiated the protective effect against chickpea collar rot caused by *S. rolfsii in planta*.^145^ The *S. rolfsii* inoculation in soil caused 100% mortality of chickpea and rot at the collar region, whereas AgNPs-treated plants under *S. rolfsii* stress grew as flourished and healthy as the plant without *S. rolfsii* challenge. The pronounced reduction of sclerotia germination was observed in the soil system after AgNPs treatment, which might explain the inhibition of collar rot by AgNPs. Similar results were reported by Guilger *et al*., revealing that the biosynthetic AgNPs based on the enzymes from *Trichoderma harzianum* effectively suppressed the sclerotia germination and controlled white mold disease caused by *S. rolfsii* in soybean.^146^ Further, the inhibitory effect of fungal-derived AgNPs on the mycelial growth of *S. rolfsii* was superior to that of chemical-derived AgNPs. Interestingly, the application of biological control using beneficial microorganisms can be a powerful solution to manage pathogens. For instance, *T. harzianum* is famous for its high production of enzymes and is widely employed as a biocontrol agent against phytopathogens. Biosynthetic Fe_2_O_3_NPs were found to stimulate the proliferation and enzymatic activity of *T. harzianum*, inhibiting the growth of the pathogen *Sclerotinia sclerotiorum*.^147^ These results indicated that biogenic Fe_2_O_3_NPs could assist the biocontrol agent to manage phytopathogens. *Phytophthora infestans* can cause late blight in potato and tomato plants, responsible for the European potato famine in the 19th century. The biosynthetic SeNPs synthesized from *Trichoderma atroviride* could trigger the defense mechanism in tomatoes to repress the late blight caused by *P. infestans*.^148^ The SeNPs-treated tomato plants showed higher depositions of H_2_O_2_, callose, and lignin, suggesting that the SeNPs triggered the immune responses in the plant. Enzymes, such as SOD, lipoxygenase, β-1,3-glucanase, and phenylalanine lyase in tomatoes, were enhanced after SeNPs treatment. These enzymes possess antimicrobial activity against pathogenic stresses. In general, bioNPs provide a tremendous potential route in fungal diseases management.

### Bacterial Disease Management

Along with fungal pathogens, bacterial pathogens also cause many plant diseases, confining the agricultural economy. Common bacterial phytopathogens are *Acidovorax* sp., *Ralstonia* sp., *Xanthomonas* sp., *Pseudomonas* sp., *Agrobacterium* sp., *Pectobacterium* sp., *Erwinia* sp., and *Dickeya* sp. Generally, these pathogens mostly survive on the plant, and they can also be found in seeds or soils. These bacteria can produce enzymes to break the plant cell wall, invade the tissues, and infect the wounds. The symptoms of plants infected by bacteria appear as wilt, crown gall, canker, spot, rot, *etc*., affecting many crops, like rice, bean, potato, tomato, maize, cabbage, fruits, and so on (Figure 5B). Recently, bioNPs have been reported as a promising candidate to control bacterial diseases. There are several standard methods to assess the antibacterial efficacy of NPs, such as agar well/disc diffusion assay, minimum inhibitory concentration (MIC), bacterial growth in a liquid medium, swarming motility assay, flow cytometric analysis, and *in planta*/*in vivo* experiment, while SEM and TEM can be used to attain bacterial morphology and ultrastructure.

*Acidovorax oryzae*, as a soil-borne bacterial pathogen, causes bacterial brown strip disease in rice. Ahmed *et al*. produced MgONPs and chitosan-magnesium (CS-Mg) nanocomposites using bacterial strain and showed that these NPs and nanocomposites exhibited substantial antibacterial activity against *A. oryzae* (Figure 6B(i)).^149,150^ Based on SEM and TEM images, the external structure of the *A. oryzae* cell was collapsed, and internal organelles were destroyed after NPs treatment (Figure 6B(ii-iii)). A similar observation was also reported by *Masum et al*., who prepared AgNPs by fruit extract to rupture the cell wall and membrane of *A. oryzae*, resulting in the leakage of nutrient and nucleic material from the cell.^151^*Ralstonia solanacearum* is another important soil-borne phytopathogen, leading to bacterial wilt disease in various plant species, that is, tomatoes, potatoes, eggplants, bananas, and groundnuts. Alam *et al*. studied the antimicrobial activity of the biosynthetic Fe_2_O_3_NPs against *R. solanacearum in planta* and reported that the severity of the disease of tomato plants caused by *R. solanacearum* was effectively mitigated after Fe_2_O_3_NPs supplement to the soil (Figure 6C).^152^ Their SEM results showed that the bacterial cells were shrivelled with the interference of Fe_2_O_3_NPs. *Xanthomonas oryzae* pv *oryzae* is one of the most disastrous pathogens, causing leaf blight diseases of rice. The AgNPs synthesized from *Azadirachta indica* leaf extract showed a higher inhibition zone (30 mm) against *X. oryzae* at 20 ppm compared with antibiotic streptocycline (21 mm) at 200 ppm.^153^ Despite low concentration, the plant-derived NPs exhibited more potent antibacterial activity than the antibiotic. Also, Ahmed *et al*. prepared AgNPs using *Bacillus cereus* to ameliorate the damage of leaf blight diseases caused by *X. oryzae* in rice.^154^*Pseudomonas syringae* can cause canker disease of fruit trees and speck of tomatoes,^155,156^ and AgNPs produced from aqueous sumac extract could mitigate the *P. syringae*-caused canker of peach trees.^156^

The modes of NPs on the antibacterial action are well-studied, involving (a) cell wall and membrane damage, (b) intracellular penetration and damage, and (c) oxidative stress.^157−159^ Meanwhile, the functional groups attached to the NPs surface also play a vital role in antibacterial activity. These functional groups from biomolecules not only enhance the antibacterial performance but also improve the biocompatibility of NPs, rendering bioNPs more competitive than chemical NPs. The mechanism regarding the improved performance of bioNPs is still obscured. However, Kumari *et al*. investigated the difference of bactericidal mechanism between biosynthetic AgNPs and chemical-developed AgNPs.^19^ The AgNPs coated with the cell-free filtrate of *Trichoderma viride* exhibited more stability and superior antibacterial property compared with chemical-developed AgNPs. Chemical-developed AgNPs aggregated after one month, while biosynthetic AgNPs were still monodispersed after six months. These outcomes could be ascribed to the different capping agents. The chemical-developed AgNPs stabilized with a weak capping agent (citrate), while the biosynthetic AgNPs stabilized with a strong one (secondary metabolites), endowing less aggregation and smaller size of biosynthetic AgNPs. The smaller size of particles led to more uptake and internalization of AgNPs. Higher internalization of biosynthetic AgNPs resulted in excessive production of ROS, potentiating the damage to the bacterial cell membrane. Apart from the property of NPs, such as size, shape, and surface charge, it should be noted that the antimicrobial efficacy of NPs also depends on the antimicrobial activity of biomolecules coated on NPs surfaces. For instance, proteins and secondary metabolites from microorganisms, such as *Trichoderma* sp.,^19^*Aspergillus* sp.,^160^*Bacillus* sp.,^161^*Streptomyces* sp.,^162^*etc*., possess proteolytic, amylolytic, chitinase, and antimicrobial activities. Polyphenols, flavonoids, terpenoids, alkaloids, and secondary metabolites from the plant have antioxidant and antimicrobial activity.^22,163,164^ Therefore, these functional materials empower bioNPs more attractive antimicrobial efficacy. Due to the vigorous bactericidal activity and hypotoxicity of the plant extract, the bioNPs can perform as an ideal agent in bacterial diseases management.

### Pest Management

Root-knot nematodes (*Meloidogyne* sp.), including *Meloidogyne incognita* and *Meloidogyne javanica*, are the most widespread and destructive pests in the agricultural field. *Meloidogyne* sp., as a soil-borne pest, infects plant roots by developing feeding sites named galls, which limit the plant absorption of water and nutrient. Kalaiselvi *et al*. produced AgNPs using latex extract of *Euphorbia tirucalli* and proved the strong nematicidal activity of the AgNPs against *M. incognita* based on *in vitro* and *in planta* experiments.^165^ Their results indicated that the plant-derived AgNPs reduced the galls caused by *M. incognita* in tomato plants and improved plant growth under the *M. incognita* stress. Similarly, Hamed *et al*. validated the nematicidal activity of bacterial-developed AgNPs against *M. javanica*.^166^ The growth of fava bean plants infected by *M. javanica* after AgNPs application was more robust than AgNO_3_ or Vydate nematicide application. In accordance with Ghareeb *et al*., the macroalga-derived AgNPs from *Cladophora glomerata* could effectively protect tomato plants against *M. javanica* attack.^167^ The nematicidal activity of AgNPs can be explained by the malfunction of various cellular mechanisms in nematodes, such as oxidative stress, ATP synthesis, and membrane permeability.^168,169^

Apart from nematode management, bioNPs can be a practical and ecofriendly approach in pest management of the stored cereal grains and pulses. The khapra beetle (*Trogoderma granarium*) is identified as one of the most destructive stored-grain pests. Almadiy *et al*. utilized harmala alkaloids from *Peganum harmala* L. seeds to synthesize AgNPs for pest management.^170^ Alkaloids are components of the plant’s defensive system, protecting the plant against pest invasion.^171^ Almadiy *et al*. found that the total harmala alkaloids (THAs) exhibited LC_95_ of 67.1 μg/cm^2^ against *T. granarium* larvae after 24 h, while the AgNPs synthesized from THAs presented LC_95_ of 12.3 μg/cm^2^, indicating that the *T. granarium* larvae were more vulnerable to the biosynthetic AgNPs.^170^ Also, *Callosobruchus maculatus* is one of the most devastative stored pulses pests, and chemical insecticides are broadly utilized to kill the pest, which can bring about grain contamination and drug resistance. Recently, Malaikozhundan *et al*. reported that the biosynthetic ZnONPs could be applied as an insecticide in *C. maculatus* management.^172,173^ The ZnONPs were synthesized from the leaf extract of *Pongamia pinnata* and the culture solution of *Bacillus thuringiensis*, respectively. Interestingly, the plant-mediated ZnONPs and bacterial-mediated ZnONPs exhibited more vigorous insecticidal activity against *C. maculatus* than chemical-mediated ZnONPs, reflecting the increment of mortality and the reduction of fecundity, hatchability, and midgut digestive enzyme activity. The leaf of *P. pinnata* contains plenty of flavonoid compounds that possess pesticidal activity. Meanwhile, *Bacillus thuringiensis* is widely applied as a biopesticide to control various insects, producing insecticidal proteins known as δ-endotoxins (cry proteins),^174^ as biomolecules, such as flavonoids and cry proteins, coating on ZnONPs can enhance the insecticidal activity of ZnONPs. However, the mechanism of the insecticidal activity of biosynthetic AgNPs is still not fully understood.

*Helicoverpa armigera* and *Spodoptera litura* are major polyphagous lepidopteran pests in agriculture and spread primarily in Asian countries, causing considerable crop damages including potato, maize, cotton, tomato, *etc*. For effective pest control and sustainable agriculture, bioNPs can be an ideal insecticide instead of chemical insecticides. Manimegalai *et al*. synthesized AgNPs by employing *Leonotis nepetifolia* plant leaves, which contain insecticidal bioorganic compounds, that is, terpenoids, phenolics, and alkaloids, and the AgNPs-treated castor leaves were applied to evaluate the antifeedant activity of the AgNPs against *H. armigera* and *S. litura*.^175^ Their results showed that the feeding inhibitions of 82% and 79% against *H. armigera* and *S. litura* were achieved after 150 ppm AgNPs treatment, suggesting that the biosynthetic AgNPs could effectively prevent crops from pest damage. After exposure to AgNPs, the larvae of *H. armigera* and *S. litura* became malformed and shrinking and failed to reach the next growth stages (Figure 6D(i,iii)). Furthermore, significant histological variations in the midgut of AgNPs-treated *H. armigera* and *S. litura* were observed, reflecting the destruction of the basement membrane (BM), brush border (Bb), and epithelial cells (EP) (Figure 6D(ii,iv)). These results were compatible with that reported by Bharani *et al*., who showed that the AgNPs from pomegranate peel extracts altered the gut physiology of *S. litura*,^176^ the gut enzymes activities of *S. litura*, including amylase, protease, lipase, and invertase reduced significantly in a dose-dependent manner, and the gut microflora and dry weight of *S. litura* reduced dramatically after AgNPs treatment. The gut microbial disruption results in a severe impact on insect physiology, providing an effective pest management route. Therefore, bioNPs can serve as an effective and ecofriendly bioagent for pest management, promoting sustainable agricultural development.

## Conclusions and Outlook

**Figure 7:**
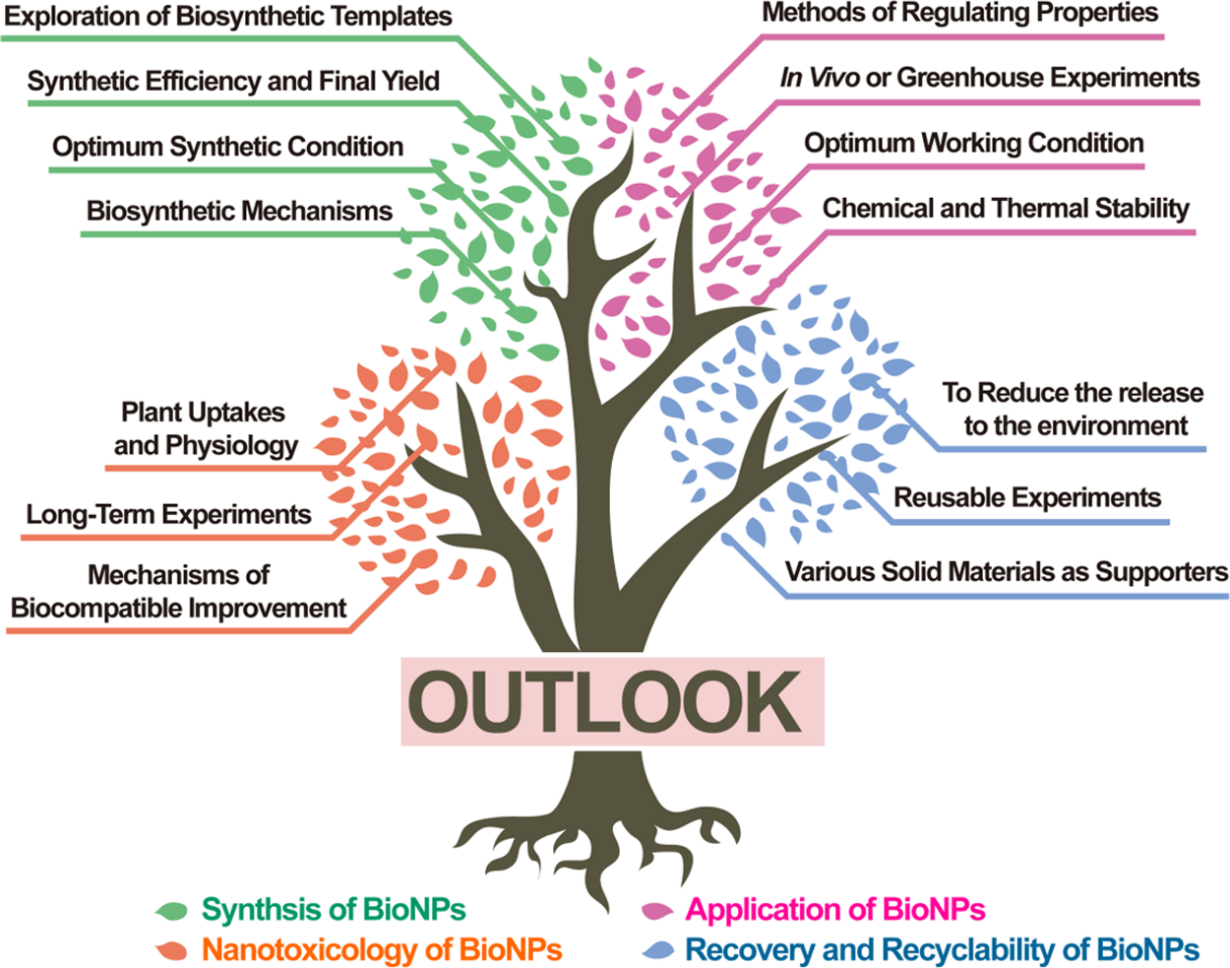
Schematic illustration of future perspectives of bioNPs in the agricultural field.

This contribution comprehensively reviews the up-to-date biosynthetic approaches of bioNPs and their applications in the scenery of sustainable agriculture. Mild synthetic conditions and biocompatible substrates award NPs various desired properties, like high performance, stability, and biocompatibility. From heavy-metal remediation, crop growth, and phytotoxicity to crop disease management, bioNPs offer an alternative avenue of advanced nanotechnology to overcome agriculture development challenges. Nevertheless, several fields require further investigations (Figure 7).

(1) The investigations of marine plants and algae for bioNPs synthesis remain largely unexplored.
(2) Genetic and chemical modifications of living species provide a protocol to synthesize the desired bioNPs.
(3) Although there are numerous successful exemplifications for bioNPs synthesis utilizing microbes, plants, and biomolecules on a laboratory scale, the synthetic efficiency and final yield of bioNPs are excluded, which are crucial indexes for industrial-scale production.
(4) Further investigations of biosynthetic mechanisms are indispensable and urgent, providing a theoretical basis for regulating bioNPs properties.
(5) Methods of accurately regulating the bioNPs properties are needed to further explore for precise application.
(6) Apart from the size and shape of bioNPs, chemical and thermal stability for an extended period should be considered as critical parameters prior to commercial applications.
(7) Optimum synthetic and working conditions are required to realize the industrial-scale production and satisfactory application of bioNPs, respectively.
(8) The effect of bioNPs on seed germination, seedling promotion, and plant disease control depends on the species of plant and environmental conditions. *In vivo* or greenhouse experiments of various plants treated with bioNPs are strongly suggested.
(9) The influences of bioNPs on plant physiology and the uptakes of bioNPs by plants as micronutrients at the molecular level need to be elaborated. It is expected that bioNPs can serve as fertilizer, improving the production of the crops.
(10) The deep insight into the improving biocompatibility of bioNPs is incomplete. Investigations on functional groups and capping materials covered on the surfaces can be an entry point to understand the nanotoxicity of bioNPs.
(11) Nanotoxicology of bioNPs on environment microorganisms, plants, and animals for long-term exposure necessitates further investigations since it is closely associated with sizes, shapes, capping agents, and surface charges of bioNPs.
(12) Recovery and recyclability of bioNPs are other significant topics. Various solid materials, like biochar, carbon nanotube, fullerene, and graphene, can support bioNPs, reducing the release of bioNPs to the environment and enhancing the recovery and recyclability of bioNPs.

Therefore, there is an enormous opportunity to explore the potential synthetic methods of bioNPs and their applications in the agricultural sector. Upon effectively surmounting these limits, bioinspired synthesis of NPs can deliver the maximum welfare to the next generations in the development of sustainable agriculture.

## Vocabulary

**Agrochemicals**: chemical products comprised of plant-protection chemicals, pesticides, fertilizers, and plant-growth hormones used in agriculture
**bioinspired synthesis**: combines biological concepts, mechanisms, and functions for the design and development of bioderived (nano)materials with various applications
**sustainable agriculture**: the goal of sustainable agriculture is to meet society’s food and textile needs in the present without compromising the ability of future generations to meet their own needs
**periplasm**: the region in a Gram-negative bacterium between the plasma membrane and an outer surrounding membrane that contains especially enzymes and a thin layer of peptidoglycan
**reactive oxygen species**: produced by enzymatic/nonenzymatic metabolic redox reactions starting with the partial reduction of oxygen to superoxide (O^2–^) or hydrogen peroxide (H_2_O_2_) followed by further secondary reactions of the products
**malondialdehyde**: formed during oxidative degeneration as a product of free oxygen radicals, which is accepted as an indicator of lipid peroxidation
**sclerotia**: compact masses of mycelium, often spherical or pellet-shaped, that range from 1 mm to 1 cm in diameter and consist of a central core of hyphae with lipid and glycogen reserves protected by a thick-walled rind
